# The macroeconomic spillovers from space activity

**DOI:** 10.1073/pnas.2221342120

**Published:** 2023-10-16

**Authors:** Luisa Corrado, Stefano Grassi, Aldo Paolillo, Edgar Silgado-Gómez

**Affiliations:** ^a^Department of Economics and Finance, University of Rome ‘Tor Vergata’, Rome 00133, Italy; ^b^Faculty of Economics and Management, Free University of Bozen-Bolzano, Bolzano 39100, Italy; ^c^Research and Macroeconomic Modelling Unit, Central Bank of Ireland, Dublin D01 F7X3, Ireland

**Keywords:** macroeconomy, space economy, growth

## Abstract

The resurgence in space activities we are witnessing may provide opportunities for new technologies to generate potential spillovers to the real economy. To address this view, we propose a macroeconomic model with endogenous growth and a space sector. The model describes the relationship between space investment and technological spillovers, which support persistent economic growth. Our estimates indicate that space activities provide growth spillovers that peak from the late 1960s to the early 1980s. Recent space activities have a much lower economic impact. Finally, extensive experiments quantify the economic relevance of our results.

## Significance

Over the past century, space activities have required the creation of ground-breaking technologies to cope with an unfavorable environment. Although there is anecdotal evidence that these advances are relevant to our lives, formal research on the impact of space missions is still scarce. In order to address the existing gap, this research examines the economic effects of space-related activities on Earth. We present an empirical assessment of the effects of space missions from the 1960s to the present day using a model in which space activity has an impact on technology. We provide evidence that space activities are likely to have produced positive economic spillovers on Earth.

On May 30, 2020, more than 50 y after the Apollo missions (1), the Falcon 9 rocket launched a new space capsule, the Crew Dragon. This mission falls under the umbrella of SpaceX, a private company founded in 2002 by Elon Musk. On the contrary, all the space missions from the Mercury (1961) to the Space Shuttle (1981 to 2011) were carried out by massive US government projects led mainly by NASA, as the large costs and risks involved made the sector generally inaccessible to private actors. The incredible range of high-tech innovations required to run the Apollo program has therefore been driven primarily by demand in the space sector: the world’s largest rocket, the world’s smallest and fastest computer, the world’s first high-speed data network, space suits, and space food (2). Most of the technology for the Apollo program had to be invented from scratch with an estimated inflation-adjusted cost of approximately $152 billion (3). From physics to chemistry, material sciences, and engineering, the pursuit of space has produced revolutionary technologies that have been translated into the industrial sector.

Today, with the decreasing costs of spacecraft development and improved remote sensing and data analytic capabilities, private space companies have undertaken more space exploration and investment activities. Since its first successful mission in 2008 (SpaceX’s Falcon 1), the private sector has entered the space industry with other milestone missions, such as Blue Origin (2016), funded by Jeff Bezos, and SpaceX’s Falcon 9 (2017). Overall, commercial space is a large and rapidly growing market, which will be worth trillions of dollars over the next decade (3).

Recent developments in space activities have shown significant changes over time in how private and public actors interact. There has been a shift from a centralized public space industry to a decentralized, competitive, arms-length sector where private actors carry more risk than before (4). These recent collaborations take the form of public–private partnerships in which public and private actors share risks and rewards.

The renaissance of space activities, which we have witnessed over the last two decades, opens the door to potentially major technological developments that may have large economic spillovers (5). These spillovers may represent an extra stimulus for global economies, (6) for a thoughtful discussion. Eventually, this new era of space activities may allow humans to settle in space in the future and enhance the sustainability of life on Earth.

Studies quantifying the economic spillovers of space activities on Earth are still lacking. To address the existing gap, we propose a two-sector real business cycle growth model that features a spillover from the space industry into new technologies. The model is estimated using Bayesian methods on US data, and we include sectoral data for the space industry. Our main finding is that space activities provide positive spillovers to the economy with different intensities over time. These intensities reach their highest values between the end of the 1960s and the beginning of the 1980s and their lowest values in the 2000s. We consider a simulation exercise that increases space production by the same amount under the high and low spillover scenarios. We find that the transmission effects on output are more than double when associated with the high space sector spillover.

Our model also provides a policy-oriented quantification of the economic impact of space sector spillovers. In particular, we assess their economic relevance by showing that space expenditure policies can provide substantial financial gains. We find that the multipliers associated with the space-related spillover of the early decades are substantial and twice those of the later decades. Exploiting the gains from space may be particularly attractive to policymakers as a possible tool to counteract the stagnant growth experienced by developed economies and deep recessions such as the COVID-19 crisis of 2020. To strengthen our argument, we provide a counterfactual analysis for the quarters immediately following the outbreak of the pandemic in order to quantify the economic relevance of the space sector spillovers in such a context. We find that the high space sector spillover in the early decades could provide a fast way to close the growth gap accumulated after the COVID-19 pandemic. On the contrary, the space sector spillover of the later decades seems to be only moderately beneficial.

The paper is connected to the literature on endogenous technological change and economic growth as refs. (7–11), and (12). The main feature of our paper is its focus on a special source of growth, namely space activity. The paper is also related to the vast and rapid development of space exploration, which opens the door to studying the consequences of past and future explorations from a microperspective and macroperspective, and treating the space economy as a field comparable to development economics, agricultural economics, information economics, resource economics, and political economy. Although space is likely to soon become a prominent item in economists’ research agenda, very few contributions have appeared: Ref. 13 deals with the provision of Earth observation from space by studying the trade-offs of organizing the activities into larger or smaller projects; ref. 14 analyzes the role of the United States in the context of globalization in accessing outer space; ref. 15 explores the impact that a tax on orbital satellites has on the long-term value of the space industry; ref. 16 provides a game-theoretical approach to the study of the removal of space debris; ref. 17 presents a thoughtful analysis of the development of the space economy by providing a characterization of the space market and of the possible government interventions to address market failures; ref. 18 discusses why space economics is an important field of research; ref. 19 discusses different types of asteroid activities, classifying them into scientific research (science), human settlement in other parts of the solar system (settlement), planetary defense (security), and mining (sales). With the exception of case studies that concentrate on returns from specific space programs or sectoral studies on the space industry, contributions that analyze the technological developments spilling over from space are still lacking. Among these exceptions, ref. 20 analyzes the economic contribution made by the space and defense industry in terms of employment, value added (contribution to GDP), sales (output), labor income, and taxes. Our contribution differs from the cited studies by building and estimating a stochastic and dynamic model, microfounded on theory.

The paper is organized as follows. The first section describes the endogenous growth model with the space sector. The Solution and Estimation section describes the dataset and the estimation strategy. The Economic Results section presents the main empirical findings with robustness checks. Finally, the Future Research section outlines potential areas for further investigation. For additional material related to the paper, we refer the reader to *SI Appendix*.

## Model

**Fig. 1.**
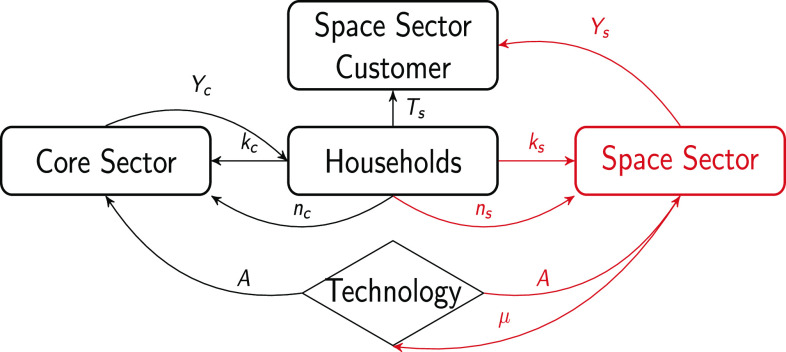
The flowchart of the economy. The arrows represent the flow of the indicated variables. $Y_{c}$ and $Y_{s}$ are the final goods produced by the firms and demanded by the households and the space sector customer; $k_{c}$ and $k_{s}$ represent the capital stocks that are rented by the households to the core sector and space firms; $n_{c}$ and $n_{s}$ are the hours of work that are supplied by the households and employed by the core sector and space firms; $T_{s}$ are the resources collected from the households to finance the demand of the space sector customer; A represents the long-term productivity component depending both on exogenous factors increasing productivity and the space sector spillover (μ).

The economy is composed at each time t by households, the core sector ($S_{c}$), and the space sector ($S_{s}$). As shown in Fig. 1 (time t subscripts are omitted for convenience), households consume the core sector goods ($Y_{c}$), and the space sector customer sets the demand for the space sector goods ($Y_{s}$).

The space sector customer is represented by the public space agency (e.g., NASA) or the private company^*^ (e.g., SpaceX) who requires the output (e.g., rockets) produced by the space sector (e.g., Boeing or SpaceX itself) to carry out space activities. The demand for space sector goods from the space sector customer is funded with the resources provided by the households through taxation ($T_{s}$).^†^

The households also supply labor ($n_{c}$, $n_{s}$) and rent capital ($k_{c}$, $k_{s}$) to the firms in the two sectors. The long-term productivity component (A) expands as a result of (i) exogenous factors that increase productivity and (ii) the space sector spillover (μ). Finally, the interest rate (R) is set by a central bank.

### Space Sector.

The space sector customer exogenously sets the demand for space sector goods, choosing the amount of expenditure in space sector production at time t ($Y_{s,t}$) and by fixing it as a share ($g_{s,t}$) of core goods production ($Y_{c,t}$):

[1] $$\begin{array}{c} \frac{Y_{s,t}}{Y_{c,t}}=g_{s,t}. \end{array}$$

The space sector share ($g_{s,t}$) follows an exogenous autoregressive (AR) process:

[2] $$\begin{array}{c} \log\left(g_{s,t}\right)=\left(1-\rho_{s}\right)\log\left(\chi \right)+\rho_{s}\log\left(g_{s,t-1}\right)+\varepsilon_{s,t}, \end{array}$$

where χ is the steady state of the space sector share; $\rho_{s}$ is the AR coefficient; and $\varepsilon_{s,t}$ is a zero mean Gaussian white noise representing the space activity shock with standard deviation (SD) equal to $\sigma_{s}$.

The supply of space sector goods is provided by the space sector that charges a flexible price ($P_{s,t}$) and is equipped with a production function. The space firm rents space capital ($k_{s,t}$) and demands labor ($n_{s,t}$) from households, taking the input prices (the wage $w_{s,t}$ and the capital rental rate $r_{k_{s},t}$) as given to maximize its nominal profits:

[3] $$\begin{array}{c} \max\left(P_{s,t}Y_{s,t}-P_{s,t}w_{s,t}n_{s,t}-P_{s,t}r_{k_{s},t}k_{s,t-1}\right), \end{array}$$

where $P_{s,t}Y_{s,t}$ are the total revenues, $P_{s,t}w_{s,t}n_{s,t}$ is the labor cost, and $P_{s,t}r_{k_{s},t}k_{s,t-1}$ is the capital rental cost.

The firm is subject to the production function:

[4] $$\begin{array}{c} Y_{s,t}=a_{z_{s},t}{\left(A_{t}n_{s,t}\right)}^{1-\alpha_{s}}{\left(k_{s,t-1}\right)}^{\alpha_{s}}, \end{array}$$

where $Y_{s,t}$ is the output of the space sector; the variable $a_{z_{s},t}$ represents the short-term fluctuations in the productivity of the space sector, which adds to the long-term productivity component $A_{t}$; $\alpha_{s}$ is the share of capital in the production function.

Space sector products are finally bought by the space sector customer, who covers their costs by levying taxes on households:

[5] $$\begin{array}{c} T_{s,t}=w_{s,t}n_{s,t}+r_{k_{s},t}k_{s,t-1}, \end{array}$$

where the labor and capital costs on the right-hand side (r.h.s.) are the same as Eq. 3 but expressed in real terms.

### Core Sector.

The core sector is symmetric to the space sector and faces an analogous maximization problem subject to a production technology that is isomorphic to the one of the space sector:

[6] $$\begin{array}{c} Y_{c,t}=a_{z_{c},t}{\left(A_{t}n_{c,t}\right)}^{1-\alpha_{c}}{\left(k_{c,t-1}\right)}^{\alpha_{c}}. \end{array}$$

The complete problem and the derivations are reported in *SI Appendix*.

### Households.

At each time t, the households choose the amount of consumption ($c_{t}$), the hours supplied to the two sectors ($n_{c,t}$ and $n_{s,t}$), the amounts of investment in the capital stocks of the two sectors ($i_{c,t}$ and $i_{s,t}$), and the bonds to hold ($b_{t}$) to maximize lifetime utility:

[7] $$\begin{array}{c} E_{0}\sum_{t=0}^{\infty }{\left(\beta \Gamma \right)}^{t}\left[\log\left(c_{t}\right)-\varphi^{c}\frac{n_{c,t}^{1+\nu_{c}}}{1+\nu_{c}}-\varphi^{s}\frac{n_{s,t}^{1+\nu_{s}}}{1+\nu_{s}}\right]. \end{array}$$

Eq. 7 describes the discounted flow of utility coming from consuming the core goods, less the disutility of supplying labor to the two sectors. The parameter β is the intertemporal discount rate which is scaled by the gross growth rate of the economy (Γ) to take into account technological progress. The parameters $\nu_{c}$ and $\nu_{s}$ determine the curvature of the labor disutility and measure the elasticity of labor supply to the wage rate. The weights $\varphi^{c}$ and $\varphi^{s}$ are scale coefficients that impose steady-state values for hours worked that are consistent with historical averages.

The households must satisfy the following budget constraint while maximizing utility:

[8] $$\begin{array}{cc} c_{t}+i_{c,t}+p_{s,t}i_{s,t}+b_{t}= & \;w_{c,t}n_{c,t}+p_{s,t}w_{s,t}n_{s,t}\\ & +r_{k_{c},t}k_{c,t-1}+p_{s,t}r_{k_{s},t}k_{s,t-1}\\ & +\frac{R_{t-1}b_{t-1}}{\pi_{c,t}}-p_{s,t}T_{s,t}, \end{array}$$

which is expressed in real terms by defining the relative price of space sector goods to core sector goods as $p_{s,t}=P_{s,t}/P_{c,t}$. In Eq. 8, the elements on the r.h.s. represent the net source of funds coming from wage income in the core sector ($w_{c,t}n_{c,t}$) and in the space sector ($p_{s,t}w_{s,t}n_{s,t}$), returns on capital rented to core firms ($r_{k_{c},t}k_{c,t-1}$) and to space firms ($p_{s,t}r_{k_{s},t}k_{s,t-1}$), and from liquidating assets represented by expiring bonds ($b_{t-1}$). Bonds are issued in nominal units in terms of the numeraire (the core good) and have a risk-free gross yield equal to $R_{t}$, so the real gross returns are given by $R_{t-1}b_{t-1}/\pi_{c,t}$, where $\pi_{c,t}$ denotes the inflation rate of the numeraire ($P_{c,t}/P_{c,t-1}$). The households also provide the space sector customer with the resources needed to finance the demand of the space sector goods ($p_{s,t}T_{s,t}$). The elements on the left-hand side (l.h.s.) show how available funds are allocated between consumption ($c_{t}$), investment in core ($i_{c,t}$) and space sector capital ($i_{s,t}$), and new bonds ($b_{t}$). Net investment in the core sector ($i_{c,t}$) and in the space sector ($i_{s,t}$) is equal to the difference between the new capital amount minus the previous period amount, net of depreciation:

[9] $$\begin{array}{c} i_{c,t}=k_{c,t}-\left(1-\delta_{k_{c}}\right)k_{c,t-1},\;\;\text{and}\;i_{s,t}=k_{s,t}-\left(1-\delta_{k_{s}}\right)k_{s,t-1}, \end{array}$$

where the parameters $\delta_{k_{c}}$ and $\delta_{k_{s}}$ are the capital depreciation rates for the core and space sectors.

### Technology.

The long-term productivity component ($A_{t}$) of production functions drives economic growth in the real variables of the model. To reflect the potential impact of space on technological progress, we allow for a spillover effect from space activity ($Y_{s,t}$) to the level of technology ($A_{t}$). We are agnostic about the direction of the spillover and test its sign (positive or negative) empirically, see the next section.

Following refs. (10) and (12), we assume that a new technology needs time to be adopted. In line with these authors, we denote existing technologies with $Z_{t}$ and adopted technologies with $A_{t}$. We assume that the number of existing technologies evolves as follows:

[10] $$\begin{array}{c} Z_{t}=\phi Z_{t-1}+\xi_{t-1}{\left({\tilde{Y}}_{s,t-1}\right)}^{\mu_{t}}, \end{array}$$

where ${\tilde{Y}}_{s,t}$ represents detrended production in the space sector $\left(Y_{s,t}/A_{t}\right)$. The parameter ϕ captures the probability that a technology will survive in the next period (not become obsolete). The term $\xi_{t}$ is a scaling factor that ensures the existence of a balanced growth path, and it is equal to $\xi_{t}=\hat{\xi }A_{t}$, where $\hat{\xi }$ is a constant that controls the steady-state growth rate. Production in the space sector (${\tilde{Y}}_{s,t-1}$) allows for a potential effect of space activity on technology. In particular, $\mu_{t}$ represents the time-varying space sector spillover, which according to Eq. 10 equals the elasticity of new technologies to production in the space sector, namely the percentage change of new technologies in response to percentage change in space production.

The law of motion of adopted technologies is given by

[11] $$\begin{array}{c} A_{t}=\left[\phi A_{t-1}+\lambda \phi \left(Z_{t-1}-A_{t-1}\right)\right]\exp\left(\varepsilon_{x,t}\right), \end{array}$$

where the variable $\varepsilon_{x,t}$ represents a zero mean Gaussian white noise reflecting exogenous movements in technology that affect growth and are not related to space. Its SD is denoted by $\sigma_{x}$. Eq. 11 implies that, at each time t, an adopted technology has a probability ϕ to survive and that a technology in the pool of unadopted technologies ($Z_{t-1}-A_{t-1}$) has a probability λ to be adopted, conditional on its survival. The adoption probability (λ) is inversely related to the average adoption lag (τ), according to the simple formula $\lambda =\frac{1}{4\tau }$. Thus, when τ increases, the probability λ decreases and adopted technologies ($A_{t}$) respond less to technologies generated by the space sector spillover ($Z_{t}$). To obtain the stationary form of Eq. 11, we divide it by $A_{t-1}$, which gives the stochastic growth rate of the economy ($x_{t}$):

[12] $$\begin{array}{c} \exp\left(x_{t}\right)\equiv \frac{A_{t}}{A_{t-1}}=\left[\phi +\lambda \phi \left({\tilde{Z}}_{t-1}-1\right)\right]\exp\left(\varepsilon_{x,t}\right), \end{array}$$

where ${\tilde{Z}}_{t-1}$ represents detrended existing technologies $\left(Z_{t-1}/A_{t-1}\right)$. The growth rate $x_{t}$ determines the balanced growth path of the real variables in the model, and its steady-state value is equal to the net growth rate of the economy, γ=log(Γ).

### Aggregation and Equilibrium.

The interest rate is set according to a simple Taylor rule:

[13] $$\begin{array}{c} R_{t}=R_{\mathrm{ss}}\pi_{c,t}^{r_{\pi }}, \end{array}$$

where $R_{\mathrm{ss}}$ denotes the steady-state gross interest rate and $r_{\pi }$ is the response of the interest rate to inflation.

The resource constraint for the core sector ensures that the amount of consumption and investment are equal to production:

[14] $$\begin{array}{c} c_{t}+i_{c,t}+p_{s,t}i_{s,t}=Y_{c,t}. \end{array}$$

The exogenous production requirement in Eq. 1 ensures that the demand of the space sector customer is equal to the supply of the space firm, thus enforcing equilibrium in the space sector. The aggregate production of the economy in real terms is given by:

[15] $$\begin{array}{c} GDP_{t}=Y_{c,t}+p_{s,t}Y_{s,t}. \end{array}$$

Finally, the evolution of the relative price of the space good is linked to the evolution of the inflation rates in the two sectors:

[16] $$\begin{array}{c} \frac{p_{s,t}}{p_{s,t-1}}=\frac{P_{s,t}/P_{c,t}}{P_{s,t-1}/P_{c,t-1}}=\frac{\pi_{s,t}}{\pi_{c,t}}. \end{array}$$

### Exogenous Processes.

The exogenous processes are AR, and they describe the evolution of the core sector short-term fluctuation in productivity ($a_{z_{c},t}$); the space sector short-term fluctuation in productivity ($a_{z_{s},t}$); and the time-varying component of the space sector spillover ($a_{\mu ,t}$). More in detail:

[17] $$\begin{array}{c} \log\left(a_{z_{c},t}\right)=\rho_{z_{c}}\log\left(a_{z_{c},t-1}\right)+\varepsilon_{z_{c},t}, \end{array}$$

[18] $$\begin{array}{c} \log\left(a_{z_{s},t}\right)=\rho_{z_{s}}\log\left(a_{z_{s},t-1}\right)+\varepsilon_{z_{s},t}, \end{array}$$

[19] $$\begin{array}{c} \log\left(a_{\mu ,t}\right)=\rho_{\mu }\log\left(a_{\mu ,t-1}\right)+\varepsilon_{\mu ,t}, \end{array}$$

where $\rho_{z_{c}}$, $\rho_{z_{s}}$, and $\rho_{\mu }$ are the AR coefficients and $\varepsilon_{z_{c},t}$, $\varepsilon_{z_{s},t}$ and $\varepsilon_{\mu ,t}$ are Gaussian white noise with SD equal to $\sigma_{z_{c}}$, $\sigma_{z_{s}}$ and $\sigma_{\mu }$.

The time-varying space sector spillover ($\mu_{t}$) appearing in Eq. 10 is

[20] $$\begin{array}{c} \mu_{t}=\mu \times a_{\mu ,t}, \end{array}$$

where μ is the time-invariant part given by the unconditional mean value.

## Solution and Estimation

The model presented in the previous section contains nonstationary variables because of endogenous growth in the level of technology. In particular, the existing and adopted technologies ($Z_{t}$ and $A_{t}$) show a stochastic trend that affects all the real variables of the model. Therefore, we transform these variables in terms of deviations from the balanced growth path to have a proper steady state. The dataset spans from 1960:Q1 to 2018:Q4 and is based on quarterly US variables: real GDP, aggregate consumption, and the industrial production index of the space industry. The time series are taken in non-demeaned growth rates to retain information on the trend. A detailed description of the data is reported in *SI Appendix*. The estimation strategy follows a standard random walk Metropolis–Hastings algorithm (RWMH) to target the posterior distribution of the model parameters.^‡^

Following common practice, some parameters are calibrated, see Table 1. In particular, the parameters for the capital share in technology are set equal to $\alpha_{c}=\alpha_{s}=0.35$ to match a labor share of income of 0.65, see Eqs. **4** and **6**. The quarterly intertemporal discount rate (β) in Eq. 7 is set at 0.991, giving an annual interest rate of 3.60% at the steady state. The quarterly capital depreciation rates are equal to $\delta_{k_{c}}=\delta_{k_{s}}=0.025$, implying an annual depreciation rate of 10%. The steady state of the space sector share ($g_{s}$) in Eq. 2 equals χ=0.56/100, matching the observed historical average.^§^ The average growth rate of the economy is calibrated to impose an annual steady-state growth rate of 1.80%, so that the quarterly growth rate is γ=0.45/100. The labor disutility curvature parameters $\nu_{c}$ and $\nu_{s}$ are set to 2.00 following the microcalibration (21). The $r_{\pi }$ reaction parameter in the Taylor rule is set to the standard value of 1.50 (22). The weights $\varphi^{c}$ and $\varphi^{s}$ are set to calibrate a ratio of hours worked in the two sectors ($n_{s}/n_{c}$) equal to the ratio of space sector production to core sector production ($Y_{s}/Y_{c}$); the calibration also imposes a relative price of the space good ($p_{s}$) normalized to 1 in steady state. Following ref. 12, we target a quarterly obsolescence rate of technologies equal to 2%, which gives a probability that the technology will not become obsolete equal to 1−0.02, so ϕ=0.98.

**Table 1.** Calibrated parameters

Full name	Symbol	Value
Hours worked in $S_{c}$	$n_{c}$	1.000
Depreciation $S_{c}$	$\delta_{k_{c}}$	0.025
Inverse Frisch elast. $S_{c}$	$\nu_{c}$	2.000
Capital share $S_{c}$	$\alpha_{c}$	0.350
Hours worked in $S_{s}$	$100\times n_{s}$	0.560
Depreciation $S_{s}$	$\delta_{k_{s}}$	0.025
Inverse Frisch elast. $S_{s}$	$\nu_{s}$	2.000
Capital share $S_{s}$	$\alpha_{s}$	0.350
Space sector share	100×χ	0.560
Discount factor	β	0.991
Interest rate rule	$r_{\pi }$	1.500
Survival rate of technologies	ϕ	0.980
Growth rate in steady state	100×γ	0.450

The table reports the parameter’s name (Full name), the associate symbol (Symbol), and the calibrated value (Value).

The prior selection of the estimated parameters is consistent with the literature on dynamic stochastic general equilibrium (DSGE) models, ref. 23 and is reported in Table 2. To allow for flexible shapes in the response of new technologies to space activity, and differently from ref. 12, we estimate the probability of adopting a new technology (λ). More precisely, we estimate the average adoption lag in years (τ), which is related to λ by the simple formula $\tau =\frac{1}{4\lambda }$, see the previous section. For τ, we then select a normal (N) prior centered around a mean value corresponding to the calibrated value in ref. 12. We do not have evidence from previous studies for the time-invariant space sector spillover (μ). As we are agnostic about the sign of the spillover, we use a uniform prior (U) with interval [−1.00,1.00]. This prior does not rule out the possibility of a negative space sector spillover (e.g., a potential misallocation of research efforts caused by space activities). Finally, for the exogenous processes, we use inverse-gamma (IG) priors for the SD and beta (B) priors for the AR coefficients.

**Table 2.** Estimation results

Symbol	Prior	Prior Mean	Prior St. Dev	Post. Mean	Post. St. Dev
τ	N	5.00	1.00	5.39	0.97
μ	U	0.00	0.58	0.30	0.09
$\rho_{z_{c}}$	B	0.50	0.20	0.99	0.00
$\rho_{z_{s}}$	B	0.50	0.20	0.49	0.21
$\rho_{s}$	B	0.50	0.20	0.96	0.01
$\rho_{\mu }$	B	0.50	0.20	0.99	0.01
$100\times \sigma_{z_{c}}$	IG	1.00	1.00	0.81	0.04
$100\times \sigma_{z_{s}}$	IG	1.00	1.00	0.99	0.69
$100\times \sigma_{s}$	IG	1.00	1.00	3.73	0.18
$100\times \sigma_{\mu }$	IG	1.00	1.00	4.64	1.42
$100\times \sigma_{x}$	IG	1.00	1.00	0.38	0.07

The table reports the estimated parameter’s symbol (Symbol), the prior shape (Prior), the prior mean and SD (Prior Mean, Prior St. Dev), and the posterior mean (Post. Mean) and SD (Post. St. Dev). B is the Beta distribution; N is the normal distribution; IG is the inverse-gamma distribution; and U is the uniform distribution.

In the two last columns, Table 2 reports the posterior estimates and the SD of the parameters. The estimated average adoption lag (τ) suggests a delay of more than 5 y between the discovery of a new technology and its adoption, see Eq. 11. For the process governing the dynamics of the spillover $\mu_{t}$, Eq. 20, we find a positive value of the time-invariant component μ (0.30) and a highly persistent time-varying process ($a_{\mu ,t}$), with an AR parameter of around 0.99. Finally, the AR parameter of the level of space activity ($\rho_{s}$) indicates a high degree of persistence.

## Economic Results

**Fig. 2.**
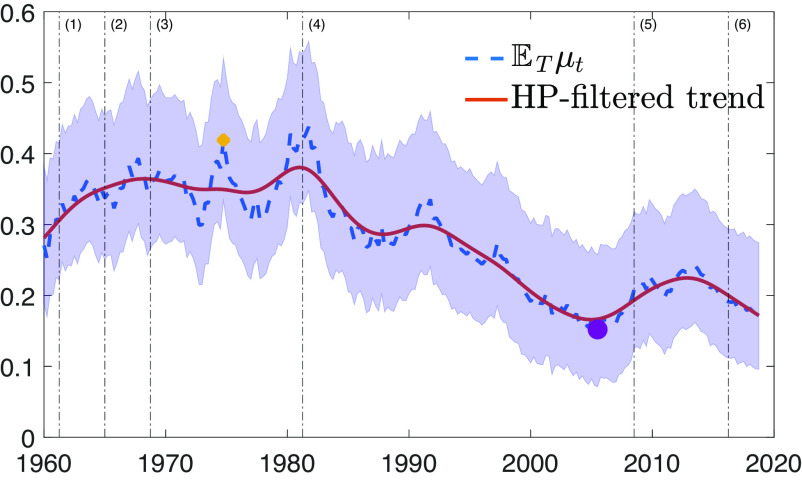
The dynamics of the space sector spillover ($E_{T}\mu_{t}$, blue dashed line) and its Hodrick–Prescott filtered trend using $\lambda^{\mathrm{HP}}=$ 16,000 (HP-filtered, red solid line). The shaded area represents the 68% credible bands related to parameter uncertainty. The six vertical dash-dotted lines represent the inaugural missions of key space programs: (1) event 1: 1961:Q2—Mercury Redstone 3; (2) event 2: 1965:Q1—Gemini 3; (3) event 3: 1968:Q4—Apollo 7; (4) event 4: 1981:Q2—Space Shuttle; (5) event 5: 2008:Q3—SpaceX’s Falcon 1; (6) event 6: 2016:Q2—Blue Origin (Amazon Company). The diamond (yellow) represents the highest value in the 1970s (μ=0.42), and the circle (purple) represents the lowest value (μ=0.15) in the 2000s.

Fig. 2 shows the estimate of $\mu_{t}$, together with credible bands. Overall, we can notice persistent fluctuations of $\mu_{t}$ over time, in line with the estimated AR parameter ($\rho_{\mu }$) of Table 2. The vertical lines report the dates of major space missions as descriptive references for the reader. The dynamics of the space sector spillover show an initial upward trend occurring during the first decades of space activity, with the three highest peaks at the end of the 1960s, mid-1970s, and early 1980s, which is in line with our a priori expectations and anecdotal evidence on the technological impact of space activities ((2) and (3)). We also detect a second downward trend starting from the early 1980s to the 2000s. In Fig. 2, we show the highest value of the 1970s, which is in the middle of the first trend ($\mu_{t}=0.42$, yellow diamond), and the lowest values of the 2000s ($\mu_{t}=0.15$, purple circle).

Overall, our estimates suggest that the space sector spillover is considerably higher in the early stages of space exploration than it is today: It is above 0.30 during the Mercury and Gemini programs (event 1 and event 2). The Apollo 7 mission (event 3) represents a major achievement in space exploration that spurred revolutionary technological improvements. After this event, there is a build-up in the space sector spillover, which pushed it to a peak of about 0.40. Similar large values in the spillover remain until the first mission of the Space Shuttle program (event 4). The plot shows that after this event, the space sector spillover declined considerably, reaching its lowest level in the 2000s, at a value around 0.15. The most recent private initiatives (event 5 and event 6) are associated with a spillover that is approximately of the same magnitude.

### IRFs.

Given the parameter estimates in Table 2 and the evolution of the space sector spillover in Fig. 2, we now study the impulse response function (IRF) of GDP to a space sector shock that unexpectedly increases production in the space sector.

The IRF is calculated by comparing the evolution of GDP in the case where the share of the space sector ($g_{s,t}$) receives an impulse from its shock ($\varepsilon_{s,t}$) to the case where it does not. Specifically, the IRF quantifies the effects that an exogenous increase in space activity (e.g., a new NASA or SpaceX mission) has on the discovery of new technologies and then on the level of GDP.^¶^ For example, space discoveries, such as new tracking systems (e.g., GPS) or more compact hardware (e.g., laptops), may drive the path of GDP to a higher trajectory, via a growth rate ($x_{t}$) that is above the steady-state value (γ).

**Fig. 3.**
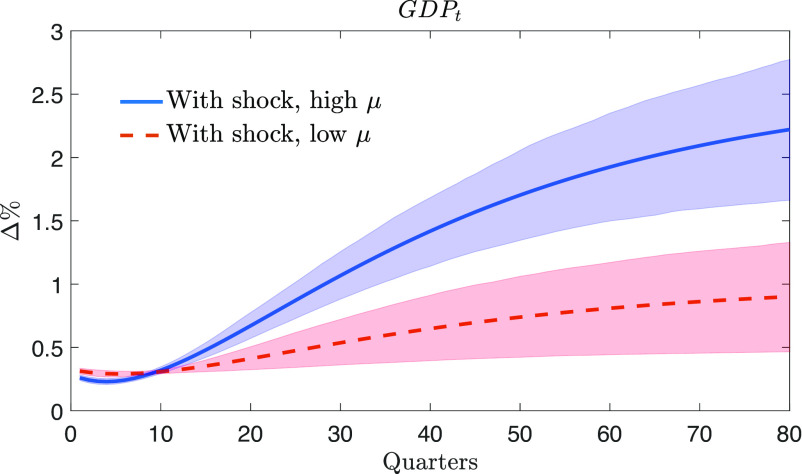
The IRF of GDP to a positive space sector shock associated with high (0.42) space sector spillover (high μ, solid blue line) and low (0.15) space sector spillover (low μ, dashed red line), expressed in percentage deviations with respect to the case where no shock realizes. The blue and red shaded areas represent credible 68% bands related to parameter uncertainty.

Fig. 3 shows the estimated IRF of GDP in two scenarios: The first one is associated with the higher space activity spillover of the mid-70s ($\mu_{t}=0.42$, blue line), while the second is associated with the lower spillover of recent years ($\mu_{t}=0.15$, red dashed line). Our analysis considers a space sector shock that hits the economy with the same size in both scenarios.^#^ We find that a space sector shock has a positive effect on GDP, and it is stronger under the high-spillover scenario of the 1970s: In this case, GDP goes up by around 2.2% after 20 y (80 quarters). Under the low-spillover scenario, the positive effect on GDP is attenuated: After 20 y, the response is only +0.9%.

**Fig. 4.**
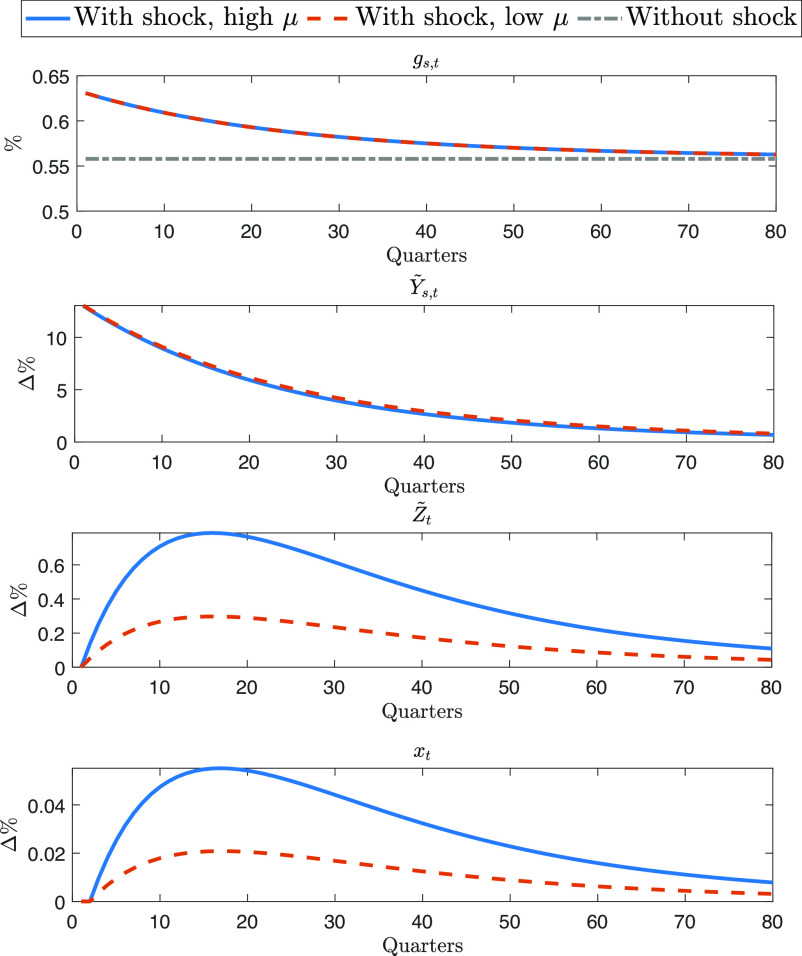
The IRFs of stationary variables to a positive space sector shock with high space sector spillover (high μ, solid blue line) and low space sector spillover (low μ, red dashed line). The gray dash-dotted line is the response when there is no shock. The upper panel reports the response of the space sector share ($g_{s,t}$), expressed in percentage points (%); the second panel reports the response of detrended space production (${\tilde{Y}}_{s,t}$) in percentage deviations from the steady state (Δ%); the third panel reports the detrended existing technologies (${\tilde{Z}}_{t}$) in percentage deviations from the steady state (Δ%); and the lower panel reports the response of the growth rate ($x_{t}$) in deviations from the steady state, expressed in percentage points (Δ%). Notice that the middle and lower plots are in percentage differences, so the gray line stays at zero and is not reported.

To gain a deeper understanding of the driving forces behind the GDP response in Fig. 3, we analyze how key economic variables respond to the same space sector shock. In the IRFs of Fig. 4, we use stationary variables compared to their steady-state values. This allows us to see how long the variables grow at a faster pace than they would do in a counterfactual trajectory without the shock. The first panel of Fig. 4 shows that the space sector shock raises the space sector share ($g_{s,t}$), as illustrated in Eq. 2. The increase in $g_{s,t}$ leads to higher production in the space sector (${\tilde{Y}}_{s,t}$), see panel two. The third panel shows that after the shock, production in the space sector generates new technologies (${\tilde{Z}}_{t}$), due to the space sector spillover ($\mu_{t}$) in Eq. 10. Finally, the fourth panel shows that the growth ($x_{t}$) in the number of adopted technologies ($A_{t}$) in Eq. 12 follows the shape of the response of new technologies (${\tilde{Z}}_{t}$). The growth rate starts from zero immediately after the shock and then reaches its peak with a delay following a hump-shaped pattern. This is consistent with the results of Table 2, where the estimated adoption lag indicates that new space discoveries need time to be usable on Earth. The prolonged positive deviation of the growth rate from the steady state explains why, in Fig. 3, the IRF of GDP continues to be upward sloping after 20 y.

### Space Spending Multipliers.

As shown in the previous subsection, a space sector shock increases the share of space production and, via the technology spillover, raises the level of aggregate production (GDP). To further corroborate this result, we compare the economic gains (increase in GDP) induced by a space sector shock at time t to the economic costs (increase in space expenditure, $p_{s,t}Y_{s,t}$). In each time period t+i after the shock, we compute a) the increases in space expenditure in the case that the shock realizes with respect to the case it does not ($\Delta p_{s,t+i}Y_{s,t+i}$) and b) the corresponding GDP gains ($\Delta GDP_{t+i}$, shown in Fig. 3). At each time horizon h, the flow of gains and expenditures are discounted and cumulated, and their ratio is taken. Following refs. (24) and (25), the expression for the cumulative space spending multiplier $M_{s,h}$ is

[21] $$\begin{array}{c} M_{s,h}=\frac{E_{t}\sum_{i=0}^{h}\left(\frac{1}{R_{t|t+i}}\right)\Delta GDP_{t+i}}{E_{t}\sum_{i=0}^{h}\left(\frac{1}{R_{t|t+i}}\right)\Delta \left(p_{s,t+i}Y_{s,t+i}\right)}. \end{array}$$

In Eq. 21, $1/R_{t|t+i}$ represents the factor used for actualizing future gains and expenses, occurring i periods ahead in the future.^‖^

**Fig. 5.**
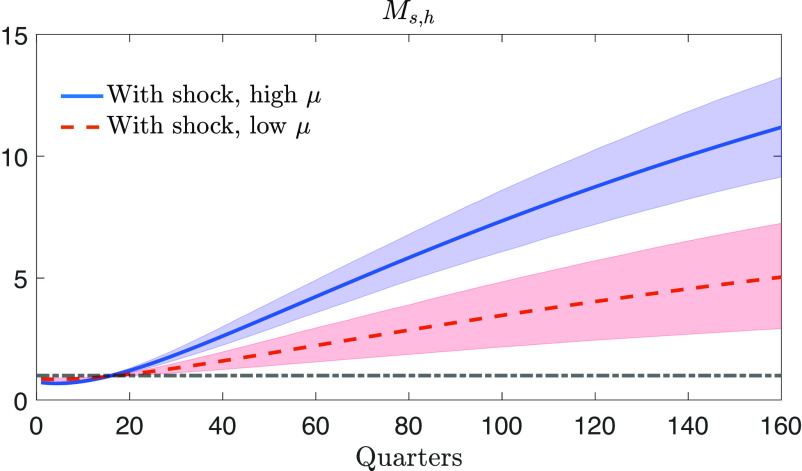
The cumulative multipliers of space expenditure ($M_{s,h}$) associated with high space sector spillover (high μ, blue solid line) and low space sector spillover (low μ, red dashed line). The gray dash-dotted line shows the level of $M_{s,h}=1$, namely the case when returns are equal to expenditure. The shaded areas represent 68% credible bands related to parameter uncertainty.

Fig. 5 shows the cumulative multipliers associated with the high and low space sector spillovers. The spending multipliers associated with the spillover of the mid-70s are much higher than those of the 2000s. Multipliers for the high space sector spillover reach values of 5 after 80 quarters and then converge in the long run to 21 (not shown in the figure for convenience). Multipliers for the low space sector spillover of recent years reach a value of 3 at horizon h=80 and then converge to 10 in the long run. Although smaller than the values of the mid-70s, multipliers still exceed unity by far.

These multipliers are larger than those often reported for more traditional types of fiscal spending (25, 26). In this regard, it should be noted that the space sector represents a very specific spending category that can lead to technological developments and technological transfers that are considerably different from those generated by ordinary spending categories (27, 28).

### Recovery.

**Fig. 6.**
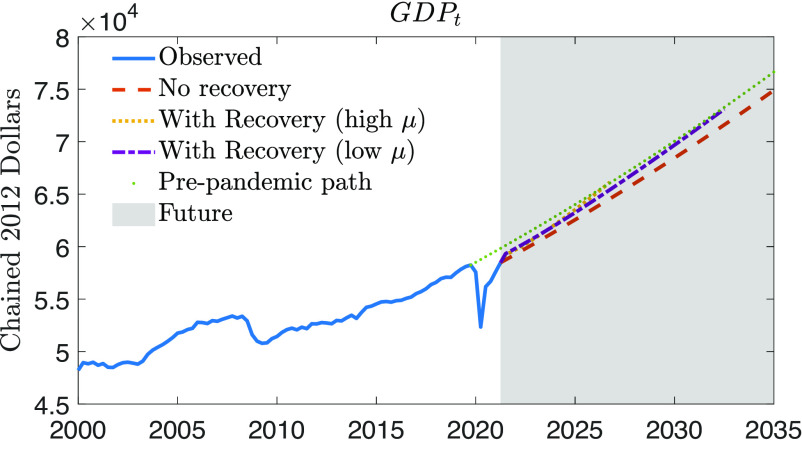
The potential effects of space expenditure on recovery after the COVID-19 pandemic. The gray area represents the forecasting period. The shock size has been chosen so as to get the space sector share to its record-high level of the sample. To reach this level, the space sector shock is scaled to have an 11 SD size. This implies raising the space sector production (${\tilde{Y}}_{s,t}$) on impact by 39% and the space sector share ($g_{s,t}$) from 0.54% to 0.76%, the maximum smoothed level of this variable found in the estimation sample.

Exploiting the gains from space activities may be particularly important in light of the recent history of slow economic growth in developed economies and the lasting scars resulting from the COVID-19 downturn. The outbreak of the pandemic has resulted in a period of economic contraction that has not yet fully ended. In this context, it is an open question whether space activity may be a useful tool to put the economy back on track. Our model can answer this question by providing forecasts for GDP, conditional on different scenarios regarding the level of production in the space sector. Fig. 6 shows the evolution of US real GDP per capita between 2000:Q1 and 2021:Q2 (solid blue line), with a large V-shape drop and partial rebound that characterize the pandemic recession of 2020. The dotted green line shows the potential growth path implied by the steady-state growth rate of the model. The figure then shows the forecast for the future, after 2021:Q2 (gray shaded area). The dashed red line represents the case in which no space spending shock ($\varepsilon_{s,t}$) is realized. The dash-dotted purple line and the dotted yellow line show the case in which a positive space sector shock occurs in 2021:Q2. These two lines refer to high and low spillovers: The dotted yellow line is associated with $\mu_{t}=0.42$, while the dash-dotted purple line is associated with $\mu_{t}=0.15$. This space sector shock could be more effective in restoring GDP to prepandemic levels. By 2027, the high spillover would completely close the growth gap, while low spillover would take longer.

### Robustness.

This section describes the main robustness checks on the estimated space sector spillover and model parameters (*SI Appendix*). The first exercise finds that our results are robust with respect to changes in key calibrated parameters in Table 1. We also study what happens when we fix some of the estimated parameters in Table 2 to alternative values and find that our model estimates provide a good fit for the data at hand. The second and third exercises replace the industrial production of the space sector with the industrial production of the information technology sector and the budgetary data summing NASA and Department of Defense spending. In both cases, we find that the spillover trajectories are qualitatively different from that in Fig. 2.

Finally, to show that our findings are not model dependent, we present additional evidence using a less restrictive model, which confirms our results.

## Future Research

The renaissance of space exploration that we are witnessing is paving the way for a number of potential technological developments that may produce significant economic spillovers. This paper addresses this view by building and estimating a two-sector general equilibrium growth model with a space sector. Our results show that space activities lead to positive spillovers to the real economy, thereby boosting productivity and long-term growth. To better quantify the macroeconomic spillovers from the space sector, we propose the following extensions for future research.

Concerning the model, a complete set of real and nominal rigidities could be incorporated, allowing the use of richer macroeconomic datasets and further extending our findings. In addition, it would be interesting to add a fully fledged public sector that allocates resources and decentralizes the production of space activities, influencing efficiency and competition among the private companies operating in the space sector (17, 29). Furthermore, the theoretical model could be extended by adding a Research and Development (R&D) sector that is microfunded. This direction has been proposed by refs. (7, 8), and (9), or more recently by refs. (10, 11), and (12) in the context of medium-scale models. This will allow us to distinguish between the relative importance of general R&D and the spillover from space activity. A better understanding of the determinants of the space activity spillover could also lead to microfunding its movements based on economic theory, for example, depending on the general level of R&D.

Concerning the data, the future availability of new and richer time series will allow researchers to estimate a variety of space activity spillovers. For example, an interesting research avenue is to estimate the space activity spillover using more granular information at a higher frequency from NASA’s budget, which is divided internally into major program areas.^**^

Recent developments show that space activities are attracting a growing number of private companies that want to exploit the opportunities for profits in this market^††^. In this environment, individual space actors have incentives to exploit the space economy quickly before others. This may potentially lead to negative technological spillovers due to risks such as major technological failures and accidents. Our model could be extended to consider a more articulated theoretical setup and a richer dataset to estimate these spillovers.

Further investigation should also identify the effects of space programs in other areas relevant to human well-being. In fact, space activities could provide far more benefits than merely boosting growth (31). In the future, public–private investment in space activities will lead to new discoveries and technological spillovers, which will ultimately improve the sustainability of life on Earth and mitigate the impact of human activities on our planet.

## Supplementary Material

Appendix 01 (PDF)

## Data Availability

Replication files data have been deposited in GitHub (https://github.com/AldoPaolillo/The-Macroeconomic-Spillovers-from-Space-Activity---PNAS).

## Footnotes

^*^Although many large private companies are involved in recent space activities, the majority of SpaceX’s early contracts were public contracts, and both NASA and the Department of Defense remain its main customers. The same applies to Blue Origin and the majority of launch companies.

^†^In both cases, the paper abstracts from the political economy or business-related determinants of space investment. Likewise, we leave the exploration of the geopolitical determinants of investment in space outside the scope of this paper.

^‡^The RWMH chain runs for 1,000,000 iterations, the first half of which is discarded as a burn-in period. The source code of all empirical results is freely available on the authors’ webpage.

^§^The value of the space sector share (χ) at the steady state is obtained by taking the historical average of the ratio of the sectoral output of the Aerospace Product and Parts Manufacturing Sector (NAICS 3364) to US nominal GDP. The value of the quarterly steady-state growth rate (γ) is chosen to match the average growth rate of US real GDP per capita over the sample.

^¶^The IRF is obtained by calculating the cumulative effect of the space sector shock on the level of GDP, which can be retrieved by summing its cyclical and trend components.

^#^The size of the shock is 3.50 SD which raises the space sector share from its steady-state level of 0.56% up to 0.63%, to simulate an increase in the level of production in the space sector of +13%.

^‖^The discount factor is given by the product of risk-free gross policy rates from t to t+i:

[22] $$\begin{array}{c} R_{t|t+i}=\prod_{j=0}^{i}R_{t+j}. \end{array}$$

Analyzing whether the multipliers may vary depending on different discount factors ($R_{t|t+i}$) between public or private space customers is left for future research.

^**^The amount of funding varies from year to year, but on average, roughly 45% of NASA’s annual budget is allocated to activities related to human spaceflight, 30% to robotic missions and scientific research, and the remaining 25% is split between aeronautics, technology development programs, staff salaries, facility management, and other overhead, see ref. 30 and https://www.planetary.org/space-policy/nasa-budget.

^††^The space-for-earth economy, which produces goods and services for use on Earth, accounted for 95% of the space sector’s $366 billion in 2019. In the future, the space-for-space economy will provide more opportunities for the production of goods and services, such as the mining of planets or asteroids for resources used to construct in-space habitats (4).
