# Supplementary material for: The macroeconomic spillovers from space activity
Source: Proc Natl Acad Sci U S A. 2023 Oct 16;120(43):e2221342120. doi: 10.1073/pnas.2221342120 (PMC10614782; doi:10.1073/pnas.2221342120)
Supplement: Supplementary file 1 — Appendix 01 (PDF) [file pnas.2221342120.sapp.pdf]

PNAS

Supporting Information for:
“The Macroeconomic Spillovers from Space Activity”

Luisa Corrado, Stefano Grassi, Aldo Paolillo, and Edgar Silgado-Gómez

Luisa Corrado
Email: luisa.corrado@uniroma2.it

This PDF file includes:

Supporting text
Tables S1-S2
Figures S1-S7
SI References

Supporting Information for "The Macroeconomic Spillovers from Space Activity"

By Luisa Corrado, Stefano Grassi, Aldo Paolillo and Edgar Silgado-Gómez

I. Model Equations

This section describes the two-sector real business cycle growth model in the main text and derives the equations resulting from the optimization problems.

Space Sector ($\mathcal{S}_s$)

As described in the Model section in the main text, the demand for space sector goods comes from a space sector customer, which can be loosely interpreted as a public agency (e.g., NASA) or a private company (e.g., SpaceX). At each time t, it is assumed that the amount of production of the space sector, $Y_{s,t}$, demanded by the space sector customer is a fraction of the production of the core sector, $Y_{c,t}$. In more detail, the amount of space sector goods over the amount of core sector goods is assumed to be equal to a time-varying share, $g_{s,t}$, which we call the space sector share. Formally:

$$\frac{Y_{s,t}}{Y_{c,t}} = g_{s,t}. \tag{S1}$$

The share of the space sector, $g_{s,t}$, follows an autoregressive (AR) process in logs:

$$\log(g_{s,t}) = (1 - \rho_s)\log(\chi) + \rho_s \log(g_{s,t-1}) + \varepsilon_{s,t}. \tag{S2}$$

In Eq. [S2], ρ_s is the AR parameter that introduces persistence to the space sector share $g_{s,t}$. The parameter χ is the steady state of the space sector share, which ensures that the mean value of this variable in the model is equal to the mean value in the sample, which is calibrated accordingly (see main text). Finally, $\varepsilon_{s,t}$ is the space sector shock, a zero mean Gaussian white noise with a standard deviation equal to σ_s. Positive (negative) shocks, $\varepsilon_{s,t}$, increase (decrease) the amount of space sector production ordered by the space sector customer.

Space firms produce goods ($Y_{s,t}$) and supply them to the space sector customer upon receiving payments ($P_{s,t}Y_{s,t}$) from it. The production of goods in the space sector is obtained by combining sector-specific labor ($n_{s,t}$) and capital ($k_{s,t-1}$) into a standard Cobb-Douglas production function. By choosing the amount of labor and capital to employ, the space firms maximize their profits, which are equal to revenues ($P_{s,t}Y_{s,t}$), minus labor costs ($P_{s,t}w_{s,t}n_{s,t}$) and capital rental costs ($P_{s,t}r_{k_s,t}k_{s,t-1}$). The optimization problem of space firms is as follows:

$$\max \left(P_{s,t}Y_{s,t} - P_{s,t}w_{s,t}n_{s,t} - P_{s,t}r_{k_s,t}k_{s,t-1}\right), \tag{S3}$$

subject to

$$Y_{s,t} = a_{z_s,t}\left(A_t n_{s,t}\right)^{1-\alpha_s}\left(k_{s,t-1}\right)^{\alpha_s}. \tag{S4}$$

In Eq. [S3], $w_{s,t}$ is the real space sector wage expressed in terms of the price in the space sector ($P_{s,t}$) and $r_{k_s,t}$ is the real rental rate of the space sector capital expressed in terms of the price

in the space sector. The term $a_{z_s,t}$ represents short-term fluctuations in productivity in the space sector, which adds to the long-term productivity component A_t. Finally, the parameter α_s is the capital share in the production function, while $1-\alpha_s$ is the labor share. The optimization problem of space firms requires taking the derivative of the profit function in Eq. [S3] with respect to the production inputs ($n_{s,t}$ and $k_{s,t-1}$) under the production function in Eq. [S4]. By equating these first-order derivatives to zero, we obtain the labor and capital demand:

- Labor demand by $\mathcal{S}_s$:

[S5] $$(1-\alpha_s)\frac{Y_{s,t}}{n_{s,t}} = w_{s,t},$$

- Capital demand by $\mathcal{S}_s$:

[S6] $$\alpha_s \frac{Y_{s,t}}{k_{s,t-1}} = r_{k_s,t}.$$

These first-order conditions state that the marginal product of labor $(1-\alpha_s)Y_{s,t}/n_{s,t}$ is equal to its marginal cost, $w_{s,t}$, and that the marginal product of capital $\alpha_s Y_{s,t}/k_{s,t-1}$ is equal to its marginal cost $r_{k_s,t}$. Due to the absence of frictions and monopolistic competition in the model, the space firms are perfectly competitive. This means that the revenues they receive ($P_{s,t}Y_{s,t}$) are equal to the costs of labor and capital. These costs are also equal to the amount that the space sector customer pays upon collecting it from households ($P_{s,t}T_{s,t}$, see the main text). Therefore, in nominal terms,

[S7] $$P_{s,t}T_{s,t} = P_{s,t}Y_{s,t} = P_{s,t}\left(w_{s,t}n_{s,t} + r_{k_s,t}k_{s,t-1}\right).$$

Given that $Y_{s,t} = T_{s,t}$, from now on we will use the terms 'space sector production' ($Y_{s,t}$) and 'space expenditure' ($T_{s,t}$) interchangeably.

Core Sector ($\mathcal{S}_c$)

The core sector firms, which represent the rest of the economy, face an optimization problem similar to the one of the space firms. Using a Cobb-Douglas production function, core sector firms choose the amount of labor ($n_{c,t}$) and capital ($k_{c,t-1}$) to produce a core sector good ($Y_{c,t}$). The core sector firms maximize their profits, which are equal to revenues ($P_{c,t}Y_{c,t}$) minus labor costs ($P_{c,t}w_{c,t}n_{c,t}$) and capital rental costs ($P_{c,t}r_{k_c,t}k_{c,t-1}$). Differently from the case of the space sector, the demand for core sector goods comes directly from the households that use these goods for consumption and to build capital stock. The optimization problem of the core sector firms is the following:

[S8] $$\max\left(P_{c,t}Y_{c,t} - P_{c,t}w_{c,t}n_{c,t} - P_{c,t}r_{k_c,t}k_{c,t-1}\right),$$

subject to

[S9] $$Y_{c,t} = a_{z_c,t}\left(A_t n_{c,t}\right)^{1-\alpha_c}\left(k_{c,t-1}\right)^{\alpha_c}.$$

In Eq. [S8], $w_{c,t}$ is the real core wage expressed in terms of the core sector price ($P_{c,t}$) and $r_{k_c,t}$ is the real rental rate of the core sector capital expressed in terms of the core sector price. The term $a_{z_c,t}$ drives short-term productivity fluctuations in the core sector, which adds to the long-term productivity component A_t. The parameter α_c is the share of capital in the production function, while $1-\alpha_c$ is the share of labor.

The optimization problem of the core sector firms requires taking the derivative of the profit function in Eq. [S8] with respect to the production inputs ($n_{c,t}$ and $k_{c,t-1}$) under the production

function in Eq. [S9]. By equating these first-order derivatives to zero, we obtain the labor and capital demand:

- Labor demand by $\mathcal{S}_c$:

[S10]
$$(1-\alpha_c)\frac{Y_{c,t}}{n_{c,t}} = w_{c,t},$$

- Capital demand by $\mathcal{S}_c$:

[S11]
$$\alpha_c \frac{Y_{c,t}}{k_{c,t-1}} = r_{k_c,t}.$$

Similarly to the space sector, the first equation states that the marginal product of labor $(1-\alpha_c)Y_{c,t}/n_{c,t}$ is equal to its marginal cost, $w_{c,t}$, and that the marginal product of capital $\alpha_c Y_{c,t}/k_{c,t-1}$ is equal to its marginal cost, $r_{k_c,t}$. Due to the absence of monopolistic competition, the profits of the core sector firms are zero.

Households

The infinitely-lived households choose consumption (c_t), the hours supplied to the two sectors ($n_{c,t}$ and $n_{s,t}$), the amounts of investment in the capital stocks of the two sectors ($i_{c,t}$ and $i_{s,t}$), and bonds (b_t) to maximize lifetime utility:

[S12]
$$\mathbb{E}_0 \sum_{t=0}^{\infty} (\beta\Gamma)^t \left[\log(c_t) - \varphi^c \frac{n_{c,t}^{1+\nu_c}}{1+\nu_c} - \varphi^s \frac{n_{s,t}^{1+\nu_s}}{1+\nu_s}\right],$$

subject to budget constraints:

[S13]
$$\begin{aligned} c_t + i_{c,t} + p_{s,t} i_{s,t} + b_t =& \frac{R_{t-1} b_{t-1}}{\pi_{c,t}} + w_{c,t} n_{c,t} + p_{s,t} w_{s,t} n_{s,t} + r_{k_c,t} k_{c,t-1} \\ &+ p_{s,t} r_{k_s,t} k_{s,t-1} - p_{s,t} T_{s,t}. \end{aligned}$$

Eq. [S12] describes the discounted flow of utility arising from the consumption of goods in the core sector, less the disutility of supplying labor to the two sectors. The parameter β is the intertemporal discount rate, which is scaled by the gross growth rate of the economy (Γ) to take into account technological progress. The parameters ν_c and ν_s determine the curvature of labor disutility and measure the elasticity of labor supply to the wage rate. The weights φ^c and φ^s are scale coefficients that impose steady state values for hours worked, which are consistent with the historical averages.

The budget constraint (Eq. [S13]) is expressed in real terms by defining the relative price of space sector goods to core sector goods as $p_{s,t} = P_{s,t}/P_{c,t}$. In Eq. [S13], the elements on the right-hand side (r.h.s.) represent the net source of funds coming from wage income in the core sector ($w_{c,t} n_{c,t}$) and in the space sector ($p_{s,t} w_{s,t} n_{s,t}$), returns on capital rented to core sector firms ($r_{k_c,t} k_{c,t-1}$) and to space firms ($p_{s,t} r_{k_s,t} k_{s,t-1}$), as well as from liquidating assets represented by bonds (b_{t-1}). Bonds carry a risk-free gross yield equal to R_t, so the real gross returns are $R_{t-1} b_{t-1}/\pi_{c,t}$, where $\pi_{c,t}$ is the inflation of the numeraire ($P_{c,t}/P_{c,t-1}$). The elements on the left-hand side (l.h.s.) show how available funds are allocated between consumption (c_t), investment in the core sector ($i_{c,t}$) and space sector capital ($i_{s,t}$), and new bonds (b_t). Net investments in the core sector ($i_{c,t}$) and in the space sector ($i_{s,t}$) are equal to the difference between the new amount of capital, minus the amount in the previous period, net of depreciation:

[S14]
$$i_{c,t} = k_{c,t} - (1-\delta_{k_c})\, k_{c,t-1}, \text{ and } i_{s,t} = k_{s,t} - (1-\delta_{k_s})\, k_{s,t-1}.$$

The parameters δ_{k_c} and δ_{k_s} are the capital depreciation rates of the core and space sectors, respectively. The households' optimization problem requires maximizing the utility function in Eq. [S12] under the budget constraint in Eq. [S13]. The solution to this constrained optimization problem gives the first-order conditions. First, the marginal utility of consumption at time t (uc_t) is given by the derivative of the utility function with respect to c_t:

[S15] $$uc_t = \frac{1}{c_t}.$$

Optimization with respect to the level of bonds gives the following first-order condition:

- Euler equation:

[S16] $$uc_t = \beta \Gamma R_t \mathbb{E}_t \frac{uc_{t+1}}{\pi_{c,t+1}}.$$

The Euler equation states that the utility of one dollar today is equal to the expected utility of one dollar tomorrow, adjusted for the intertemporal discount rate (β), technological progress (Γ), and the expected real interest rate ($R_t/\pi_{c,t+1}$).

Taking the derivatives with respect to hours of work ($n_{c,t}$ and $n_{s,t}$) gives the following two conditions:

- Labor supply to $\mathcal{S}_c$:

[S17] $$\varphi^c n_{c,t}^{\nu_c} = w_{c,t} uc_t,$$

- Labor supply to $\mathcal{S}_s$:

[S18] $$\varphi^s n_{s,t}^{\nu_s} = p_{s,t} w_{s,t} uc_t.$$

These conditions determine the amount of labor supplied to firms in both sectors and imply that the marginal disutilities of work ($\varphi^c n_{c,t}^{\nu_c}$ and $\varphi^s n_{s,t}^{\nu_s}$) are equal to the marginal utilities provided by an extra amount of money earned ($w_{c,t} uc_t$ and $p_{s,t} w_{s,t} uc_t$).

Taking the derivatives with respect to capital ($k_{c,t}$ and $k_{s,t}$), we obtain the following:

- Capital supply to $\mathcal{S}_c$:

[S19] $$uc_t = \beta \Gamma \mathbb{E}_t uc_{t+1} \left[1 - \delta_{k_c} + r_{k_c,t+1}\right],$$

- Capital supply to $\mathcal{S}_s$:

[S20] $$p_{s,t} uc_t = \beta \Gamma \mathbb{E}_t p_{s,t+1} uc_{t+1} \left[1 - \delta_{k_s} + r_{k_s,t+1}\right].$$

These conditions determine the optimal amount of capital supply to firms in the core and space sectors. By replacing the Euler equation (Eq. [S16]) in the capital supply functions (Eq. [S19] and Eq. [S20]), one gets that the real returns of the two capital inputs ($1 - \delta_{k_c} + r_{k_c,t+1}$ and $1 - \delta_{k_s} + r_{k_s,t+1}$) are equal to the real return of the risk-free bonds ($R_t/\pi_{c,t+1}$).

Technology

The law of motion of existing technologies is the following:

$$Z_t = \phi Z_{t-1} + \hat{\xi} A_{t-1} \left(\frac{Y_{s,t-1}}{A_{t-1}}\right)^{\mu_t},$$

from which we obtain:

$$Z_t = \phi Z_{t-1} + \xi_{t-1} \left(\tilde{Y}_{s,t-1}\right)^{\mu_t}, \tag{S21}$$

where $\xi_{t-1} = \hat{\xi} A_{t-1}$ and $\tilde{Y}_{s,t-1} = Y_{s,t-1}/A_{t-1}$. Given the level of existing technologies, Z_t, the law of motion of adopted technologies (A_t) is represented as follows:

$$A_t = [\phi A_{t-1} + \lambda\phi (Z_{t-1} - A_{t-1})] \exp(\varepsilon_{x,t}). \tag{S22}$$

Eq. [S22] shows that the level of the technology adopted at time t, A_t, depends on the number of adopted technologies that survive the previous period, ϕA_{t-1}, on the number of technologies that survive and are adopted from the pool of unadopted technologies, $\lambda\phi(Z_{t-1} - A_{t-1})$, and on an exogenous disturbance that represents factors that influence technological growth, which are not related to space ($\varepsilon_{x,t}$). As stated in the main text, λ is the probability that a technology from the pool of unadopted technologies will be adopted and is linked to the adoption lag in terms of years (τ) in the following way:

$$\tau = \frac{1}{4\lambda}.$$

Aggregation and Equilibrium

The model is closed with a simple interest rate rule that responds to inflation:

$$R_t = R_{ss} \pi_{c,t}^{r_\pi}.$$

The resource constraint of the economy ensures that the level of core sector production is equal to the amount of general consumption (c_t), investment in the core sector ($i_{c,t}$) and investment in the space sector ($i_{s,t}$):

$$c_t + i_{c,t} + p_{s,t} i_{s,t} = Y_{c,t}. \tag{S23}$$

Finally, the exogenous production requirement in Eq. [S1] ensures that the demand of the space sector customers is equal to the supply of space firms. Additional variables are defined for convenience. The nominal wage inflation in the core sector and the space sector ($\omega_{c,t}$ and $\omega_{s,t}$) is defined as follows:

$$\omega_{c,t} = \frac{w_{c,t}}{w_{c,t-1}} \pi_{c,t}, \quad \text{and} \quad \omega_{s,t} = \frac{w_{s,t}}{w_{s,t-1}} \pi_{s,t}.$$

II. Stationarizing the model

As stated in the main text, the model features stochastic growth (x_t) in the number of existing technologies (Z_t) and the adopted technologies (A_t). The following variables reflect the resulting stochastic trend and are proportional to A_t along the balanced growth path: core sector production ($Y_{c,t}$); space sector production ($Y_{s,t}$); consumption (c_t); core capital ($k_{c,t}$); space capital ($k_{s,t}$); gross domestic product (GDP_t); real core sector wages ($w_{c,t}$); real space sector wages ($w_{s,t}$); the inverse of the marginal utility of consumption ($1/uc_t$); and bonds (b_t). To stationarize the model around the balanced growth path, the non-stationary variables are rewritten as the product of their detrended components (denoted with the tilde sign) and the number of adopted technologies (A_t). Concretely:

$$\begin{aligned} &[Z_t \quad Y_{c,t} \quad Y_{s,t} \quad c_t \quad k_{c,t} \quad k_{s,t} \quad GDP_t \quad w_{c,t} \quad w_{s,t} \quad 1/uc_t \quad b_t] = \\ &[\tilde{Z}_t \quad \tilde{Y}_{c,t} \quad \tilde{Y}_{s,t} \quad \tilde{c}_t \quad \tilde{k}_{c,t} \quad \tilde{k}_{s,t} \quad \widetilde{GDP}_t \quad \tilde{w}_{c,t} \quad \tilde{w}_{s,t} \quad 1/\widetilde{uc}_t \quad \tilde{b}_t] \times A_t. \end{aligned} \tag{S24}$$

After rewriting the equations in this form, the model relationships can be reformulated in terms of stationary variables and the stochastic growth rate, so that the variables have a proper steady state, and the model can be solved.[1]

The derivations are the following:

- Existing technologies, from Eq. [S21]:

$$Z_t = \tilde{Z}_t A_t = \phi Z_{t-1} + \hat{\xi} A_{t-1} \left(\tilde{Y}_{s,t-1}\right)^{\mu_t} = \phi \tilde{Z}_{t-1} A_{t-1} + \hat{\xi} A_{t-1} \left(\tilde{Y}_{s,t-1}\right)^{\mu_t},$$

so that

$$\tilde{Z}_t \exp(x_t) = \phi \tilde{Z}_{t-1} + \hat{\xi} \left(\tilde{Y}_{s,t-1}\right)^{\mu_t}, \qquad \text{[S25]}$$

where $\exp(x_t) \equiv \frac{A_t}{A_{t-1}}$.

- From Eq. [S22], it is possible to rewrite the expression for the rate of growth of the economy (x_t) in stationary form:

$$\exp(x_t) \equiv \frac{A_t}{A_{t-1}} = \left[\phi + \lambda\phi \left(\tilde{Z}_{t-1} - 1\right)\right] \exp(\varepsilon_{x,t}). \qquad \text{[S26]}$$

- Production in the space sector, from Eq. [S4]:

$$\begin{aligned} Y_{s,t} &= \tilde{Y}_{s,t} A_t \\ &= a_{z_s,t} \left(A_t n_{s,t}\right)^{1-\alpha_s} \left(k_{s,t-1}\right)^{\alpha_s} \\ &= a_{z_s,t} \left(A_t n_{s,t}\right)^{1-\alpha_s} \left(\tilde{k}_{s,t-1} A_{t-1}\right)^{\alpha_s}, \end{aligned} \qquad \text{[S27]}$$

so that

$$\tilde{Y}_{s,t} = a_{z_s,t} \left(n_{s,t}\right)^{1-\alpha_s} \left(\tilde{k}_{s,t-1} \frac{1}{\exp(x_t)}\right)^{\alpha_s}.$$

- Production in the core sector, from Eq. [S9]:

$$\begin{aligned} Y_{c,t} &= \tilde{Y}_{c,t} A_t \\ &= a_{z_c,t} \left(A_t n_{c,t}\right)^{1-\alpha_c} \left(k_{c,t-1}\right)^{\alpha_c} \\ &= a_{z_c,t} \left(A_t n_{c,t}\right)^{1-\alpha_c} \left(\tilde{k}_{c,t-1} A_{t-1}\right)^{\alpha_c}, \end{aligned} \qquad \text{[S28]}$$

so that

$$\tilde{Y}_{c,t} = a_{z_c,t} \left(n_{c,t}\right)^{1-\alpha_c} \left(\tilde{k}_{c,t-1} \frac{1}{\exp(x_t)}\right)^{\alpha_c}.$$

- Space sector labor demand, from Eq. [S5]:

$$(1-\alpha_s) \tilde{Y}_{s,t} A_t = \tilde{w}_{s,t} A_t n_{s,t}, \qquad \text{[S29]}$$

[1] The auxiliary variables $i_{c,t}$, $i_{s,t}$ and $T_{s,t}$ also reflect the same stochastic trend. Given their immediate relationships in terms of the variables above, Eq. [S14] and Eq. [S7], we do not report them in the vector of Eq. [S24].

so that

$$(1-\alpha_s)\tilde{Y}_{s,t} = \tilde{w}_{s,t} n_{s,t}.$$

- Core sector labor demand, from Eq. [S10]:

[S30] $$(1-\alpha_c)\tilde{Y}_{c,t} A_t = \tilde{w}_{c,t} A_t n_{c,t},$$

so that

$$(1-\alpha_c)\tilde{Y}_{c,t} = \tilde{w}_{c,t} n_{c,t}.$$

- Space sector capital demand, from Eq. [S6]:

[S31] $$\alpha_s \tilde{Y}_{s,t} A_t = r_{k_s,t} \tilde{k}_{s,t-1} A_{t-1},$$

so that

$$\alpha_s \tilde{Y}_{s,t} = r_{k_s,t} \tilde{k}_{s,t-1} \frac{1}{\exp(x_t)}.$$

- Core sector capital demand, from Eq. [S11]:

[S32] $$\alpha_c \tilde{Y}_{c,t} A_t = r_{k_c,t} \tilde{k}_{c,t-1} A_{t-1},$$

so that

$$\alpha_c \tilde{Y}_{c,t} = r_{k_c,t} \tilde{k}_{c,t-1} \frac{1}{\exp(x_t)}.$$

- Resource constraint, considering Eq. [S14] and Eq. [S23], we get:

[S33] $$\begin{aligned}&\tilde{c}_t A_t + \tilde{k}_{c,t} A_t - (1-\delta_{k_c})\, \tilde{k}_{c,t-1} A_{t-1} + p_{s,t} \tilde{k}_{s,t} A_{t-1} - (1-\delta_{k_s})\, p_{s,t} \tilde{k}_{s,t-1} A_{t-1} \\ &= \tilde{Y}_{c,t} A_t,\end{aligned}$$

so that

$$\tilde{c}_t + \tilde{k}_{c,t} - (1-\delta_{k_c}) \frac{\tilde{k}_{c,t-1}}{\exp(x_t)} + p_{s,t} \tilde{k}_{s,t} - (1-\delta_{k_s})\, p_{s,t} \frac{\tilde{k}_{s,t-1}}{\exp(x_t)} = \tilde{Y}_{c,t}.$$

- Space sector share, from Eq. [S1]:

$$\frac{\tilde{Y}_{s,t} A_t}{\tilde{Y}_{c,t} A_t} = g_{s,t},$$

so that

$$\frac{\tilde{Y}_{s,t}}{\tilde{Y}_{c,t}} = g_{s,t}.$$

- Budget constraint, from Eq. [S13]:

$$\begin{aligned}&\tilde{k}_{c,t}A_t + p_{s,t}\tilde{k}_{s,t}A_t + \tilde{c}_tA_t + \tilde{b}_tA_t\\&= \frac{R_{t-1}\tilde{b}_{t-1}A_{t-1}}{\pi_{c,t}} + \tilde{w}_{c,t}A_tn_{c,t} + p_{s,t}\tilde{w}_{s,t}A_tn_{s,t}\\&+ \tilde{k}_{c,t-1}A_{t-1}\left(1-\delta_{k_c}+r_{k_c,t}\right)\\&+ p_{s,t}\tilde{k}_{s,t-1}A_{t-1}\left(1-\delta_{k_s}+r_{k_s,t}\right) - p_{s,t}\tilde{T}_{s,t}A_t,\end{aligned}$$

so that

$$\begin{aligned}\tilde{k}_{c,t} + p_{s,t}\tilde{k}_{s,t} + \tilde{c}_t + \tilde{b}_t =& \frac{R_{t-1}\tilde{b}_{t-1}}{\pi_{c,t}}\frac{1}{\exp(x_t)} + \tilde{w}_{c,t}n_{c,t} + p_{s,t}\tilde{w}_{s,t}n_{s,t}+\\&\frac{\tilde{k}_{c,t-1}}{\exp(x_t)}\left(1-\delta_{k_c}+r_{k_c,t}\right)\\&+ p_{s,t}\frac{\tilde{k}_{s,t-1}}{\exp(x_t)}\left(1-\delta_{k_s}+r_{k_s,t}\right) - p_{s,t}\tilde{T}_{s,t}.\end{aligned}$$

- Marginal utility of consumption, from Eq. [S15]:

$$\frac{\tilde{uc}_t}{A_t} = \frac{1}{\tilde{c}_tA_t},$$

so that

$$\tilde{uc}_t = \frac{1}{\tilde{c}_t}.$$

- Euler equation, from Eq. [S16]:

$$\frac{\tilde{uc}_t}{A_t} = \beta\Gamma R_t\mathbb{E}_t\frac{\tilde{uc}_{t+1}}{A_{t+1}\pi_{c,t+1}},$$

so that

$$\tilde{uc}_t = \beta\Gamma R_t\mathbb{E}_t\left[\frac{\tilde{uc}_{t+1}}{\pi_{c,t+1}}\frac{1}{\exp(x_{t+1})}\right].$$

- Labor supply to core sector firms ($\mathcal{S}_c$), from Eq. [S17]:

[S34] $$\varphi^c n_{c,t}^{\nu_c} = \tilde{w}_{c,t}A_t\tilde{uc}_t\frac{1}{A_t},$$

so that

$$\varphi^c n_{c,t}^{\nu_c} = \tilde{w}_{c,t}\tilde{uc}_t.$$

- Labor supply to space sector firms ($\mathcal{S}_s$), from Eq. [S18]:

[S35] $$\varphi^s n_{s,t}^{\nu_s} = p_{s,t}\tilde{w}_{s,t}A_t\tilde{uc}_t\frac{1}{A_t},$$

so that

$$\varphi^s n_{s,t}^{\nu_s} = p_{s,t}\tilde{w}_{s,t}\tilde{uc}_t.$$

- Capital supply to core sector firms ($\mathcal{S}_c$), from Eq. [S19]:

$$\frac{\tilde{uc}_t}{A_t} = \beta\Gamma\mathbb{E}_t\frac{\tilde{uc}_{t+1}}{A_{t+1}}\left[1-\delta_{k_c}+r_{k_c,t+1}\right],$$

so that

$$\tilde{uc}_t = \beta\Gamma\mathbb{E}_t\frac{\tilde{uc}_{t+1}}{\exp(x_{t+1})}\left[1-\delta_{k_c}+r_{k_c,t+1}\right].$$

- Capital supply to space sector firms ($\mathcal{S}_s$), from Eq. [S20]:

$$p_{s,t}\frac{\tilde{uc}_t}{A_t} = \beta\Gamma\mathbb{E}_t p_{s,t+1}\frac{\tilde{uc}_{t+1}}{A_{t+1}}\left[1-\delta_{k_s}+r_{k_s,t+1}\right],$$

so that

$$p_{s,t}\tilde{uc}_t = \beta\Gamma\mathbb{E}_t p_{s,t+1}\frac{\tilde{uc}_{t+1}}{\exp(x_{t+1})}\left[1-\delta_{k_s}+r_{k_s,t+1}\right].$$

- We define nominal wage inflation ($\omega_{c,t}$) in the core sector ($\mathcal{S}_c$):

[S36] $$\omega_{c,t} = \frac{w_{c,t}}{w_{c,t-1}}\pi_{c,t} = \frac{\tilde{w}_{c,t}}{\tilde{w}_{c,t-1}}\exp(x_t)\pi_{c,t}.$$

- And similarly, we define nominal wage inflation ($\omega_{s,t}$) in the space sector ($\mathcal{S}_s$):

[S37] $$\omega_{s,t} = \frac{w_{s,t}}{w_{s,t-1}}\pi_{c,t} = \frac{\tilde{w}_{s,t}}{\tilde{w}_{s,t-1}}\exp(x_t)\pi_{c,t}.$$

III. Steady state

This section derives the deterministic steady state of the variables in the stationarized model. First, we impose that the steady state rate of growth of the economy (x_t) is equal to the parameter γ, or equivalently, that the gross rate, $\exp(x)$, is equal to the parameter Γ:

$$x = \gamma, \quad \text{and} \quad \exp(x) = \Gamma = \exp(\gamma).$$

The law of motion of technology growth, Eq. [S26], gives the steady state for existing technologies ($\tilde{Z}$):

$$\tilde{Z} = \frac{1}{\lambda\phi}\left(\lambda\phi + \Gamma - \phi\right).$$

The capital supply equations, Eq. [S19] and Eq. [S20], give the steady state values of the rental rates of capital in the two sectors:

[S38] $$r_{k_c} = (1/\beta - 1 + \delta_{k_c}), \quad \text{and} \quad r_{k_s} = (1/\beta - 1 + \delta_{k_s}).$$

Inflation rates are zero in the steady state and therefore the nominal wage inflation rates described in Eq. [S36] and Eq. [S37] are equal to the growth rates of real wages:

$$\pi_c = 1, \quad \pi_s = 1, \quad \omega_c = 1+\gamma, \quad \omega_s = 1+\gamma.$$

Using Eq. [S2], the space sector share is equal to the mean of its autoregressive process:

$$g_s = \chi.$$

The amount of hours worked in the two sectors is imposed according to the ratio observed in the data, and set equal to the two parameters n_c^{ss} and n_s^{ss}:

$$n_c = n_c^{ss}, \quad \text{and} \quad n_s = n_s^{ss},$$

where n_c^{ss} is normalized to $n_c^{ss} = 1$ and n_c^{ss} to $n_s^{ss} = 0.56/100$ (the weight of the space sector in the economy, see the main document). To ensure that the equilibrium value of hours worked is equal to n_c^{ss} and n_s^{ss}, the labor disutility weights φ^c and φ^s are calibrated accordingly (see below).

Using the capital demand conditions, Eq. [S32] and Eq. [S31], together with the steady state values for the rental rates of capital, Eq. [S38], it is possible to find the ratios of core sector capital and space sector capital to output (denoted, respectively, with ζ_0 and ζ_1):

$$\zeta_0 \equiv \frac{\tilde{k}_c}{\tilde{Y}_c} = \frac{\Gamma\alpha_c}{(1/\beta - 1 + \delta_{k_c})}, \quad \zeta_1 \equiv \frac{\tilde{k}_s}{\tilde{Y}_s} = \frac{\Gamma\alpha_s}{(1/\beta - 1 + \delta_{k_s})}.$$

Putting these expressions in the production functions of the two sectors, Eq. [S28] and Eq. [S27], it is possible to find the levels of output:

$$\tilde{Y}_c = \left(\frac{\zeta_0}{\Gamma}\right)^{\frac{\alpha_c}{(1-\alpha_c)}} n_c, \text{ and } \tilde{Y}_s = \left(\frac{\zeta_1}{\Gamma}\right)^{\frac{\alpha_s}{(1-\alpha_s)}} n_s.$$

Using the expression for the ratios of capital to output, ζ_0 and ζ_1, the capital stocks are equal to:

$$\tilde{k}_c = \zeta_0\tilde{Y}_c, \quad \text{and} \quad \tilde{k}_s = \zeta_1\tilde{Y}_s.$$

Substituting $\tilde{Y}_s$ into Eq. [S25], the constant for the creation of new knowledge in Eq. [S21] is then pinned down by the following expression:

$$\hat{\xi} = \frac{\tilde{Z}(\Gamma - \phi)}{\tilde{Y}_s^{\mu}},$$

where μ is the steady state for the space sector spillover μ_t, as described in the main text. The relative price of the space sector goods compared to the core sector goods (p_s) is fixed at unity, that is, $p_s = P_s/P_c = 1$. By dividing the resource constraint in Eq. [S33] by the core sector production $\tilde{Y}_c$ and using the previously defined ratios of capital to production (ζ_0 and ζ_1), we obtain the ratio of consumption to core sector production (ζ_2):

$$\zeta_2 \equiv \frac{\tilde{c}}{\tilde{Y}_c} = 1 + \frac{\zeta_0}{\Gamma}\left(1 - \delta_{k_c} - \Gamma\right) + \frac{\chi\zeta_1}{\Gamma}\left(1 - \delta_{k_s} - \Gamma\right).$$

This implies that consumption and the marginal utility of consumption are given by:

$$\tilde{c} = \zeta_2\tilde{Y}_c, \quad \text{and} \quad \tilde{uc} = \frac{1}{\tilde{c}}.$$

By equating labor supply, Eq. [S34] and Eq. [S35], and labor demand, Eq. [S30] and Eq. [S29], in the two sectors, we get the labor disutility parameters:

$$\varphi^c = \frac{1}{n_c^{1+\nu_c}}\left[\frac{(1-\alpha_c)}{\zeta_2}\right], \quad \text{and} \quad \varphi^s = \frac{1}{n_s^{1+\nu_s}}\left[\chi\frac{(1-\alpha_s)}{\zeta_2}\right].$$

From the labor supply schedules, Eq. [S34] and Eq. [S35], we obtain the wage rates:

$$\tilde{w}_c = \frac{\varphi^c n_c^{\nu_c}}{\tilde{u}_{cc}}, \qquad \tilde{w}_s = \frac{\varphi^s n_s^{\nu_s}}{p_s \tilde{u}_{cc}}.$$

IV. Data description

The measurement equations connect the observed series to their model counterparts. This allows us to represent the system as a state-space model where the unobserved components (model variables) are coupled with the observed ones (data series) and to estimate the model (see Herbst and Schorfheide, 2015). The measurement equations are as follows:

$$\begin{aligned}\Delta GDP_t^{data} &= \log(\widetilde{GDP}_t) - \log(\widetilde{GDP}_{t-1}) + x_t,\\ \Delta c_t^{data} &= \log(\tilde{c}_t) - \log(\tilde{c}_{t-1}) + x_t,\\ \Delta Y_{s,t}^{data} &= \log(\tilde{Y}_{s,t}) - \log(\tilde{Y}_{s,t-1}) + x_t.\end{aligned}$$

The observed variables for the estimation of the model are real quantities (GDP, consumption), plus a variable needed to measure the level of activity in the space sector (industrial production). On the l.h.s., we have denoted the observed data series as ΔGDP_t^{data}, Δc_t^{data}, and $\Delta Y_{s,t}^{data}$. On the r.h.s, we denote the corresponding model variables. Note that the observed trending variables (GDP, consumption, and space sector industrial production) are linked to the percentage difference of their stationarized versions ($\widetilde{GDP}_t$, $\tilde{c}_t$ and $\tilde{Y}_{s,t}$), plus the stochastic growth rate (x_t).

Gross domestic product

Real gross domestic product (GDP) is retrieved from the U.S. Bureau of Economic Analysis series GDPC1. The series is seasonally adjusted and expressed in billions of chained 2012 dollars. The GDP is divided by the Civilian Noninstitutional Population (series CNP16OV from the U.S. Bureau of Labor Statistics) to transform it in per capita terms. As shown in the measurement equations, the series is connected to the model using log differences. These rates are not demeaned, so information on growth is retained. Link: `https://fred.stlouisfed.org/series/GDPC1`.

Consumption

Aggregate real consumption in billions of chained 2012 dollars is provided by the U.S. Bureau of Economic Analysis in the PCECC96 series. The series is seasonally adjusted and is divided by the Population Level (CNP16OV) to get per capita consumption. As for GDP, the growth rates are not demeaned.
Link: `https://fred.stlouisfed.org/series/PCECC96`.

Space Sector Production

Data on space sector production are obtained from the Board of Governors of the Federal Reserve System in the IPG3364S series. The series represents an index of industrial production of Aerospace Sector Product and Parts, and it is seasonally adjusted and taken in non-demeaned growth rates.
Link: `https://fred.stlouisfed.org/series/IPG3364S`

V. Robustness

This section reports four robustness checks.[2] The first exercise changes some key estimated and calibrated parameters and checks the model performance. To check whether the model tells us something about the space sector in particular, the second and third exercises in turn replace the industrial production of the space sector with (i) the industrial production of the information technology sector and (ii) the budgetary data summing NASA and Department of Defense spending.

The fourth exercise provides an estimate of a less restrictive model (Structural Vector Autoregressive model), which confirms our results.

A. Estimated and Calibrated Parameters

1) As a first exercise, we study if our economic results on μ_t change when we change the persistence of the space activity spillover, ρ_μ. Specifically, Figure S1 compares the results for μ_t when $\rho_\mu = 0.99$, our estimate, with two alternative values: one less persistent $\rho_\mu = 0.50$ and the second that gives a non persistent process $\rho_\mu = 0$. In Figure S1 we show that as ρ_μ increases from zero to our estimate suggested by the data at hand, the dynamics of the space sector spillover moves from being a highly noisy process without trends over time, to a persistent process with well visible trends over time. The visual results of Figure S1 are further validated using a goodness of fit measure that indicates how well the model does. The measure we use is the log marginal data density (log MDD) calculated by Dynare, see Adjemian et al. (2022). The log MDD, also called the log marginal likelihood of the data, is a popular Bayesian comparison criterion that evaluates the goodness of fit of one specific model with the data at hand, see Koop (2003) and Geweke (2005). In Figure S2 we report the log MDD for the three models. As the figure shows, the log MDD gives the highest value in the case of our benchmark model. The other two models have a lower log MDD which implies a worse performance with respect to our specification, with the model and data at hand.

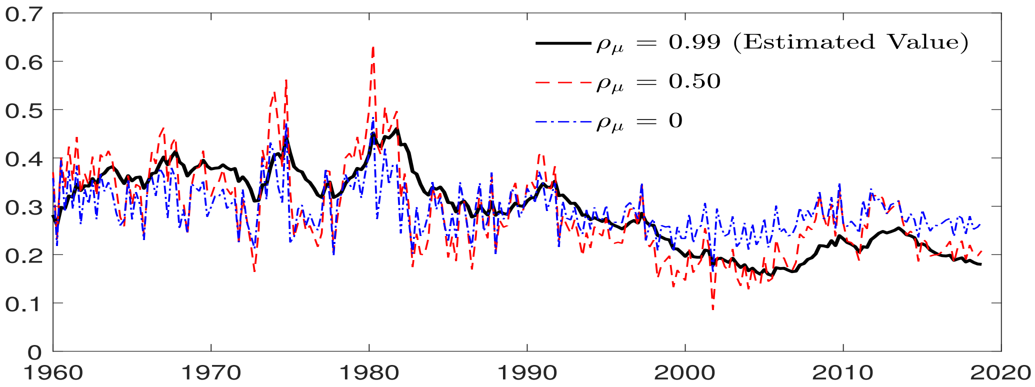

FIGURE S1. THE FIGURE REPORTS THE ESTIMATED μ_t GIVEN DIFFERENT VALUES OF ρ_μ. THE SOLID BLACK LINE IS THE ESTIMATED μ_t WITH $\rho_\mu = 0.99$. THE DASHED RED LINE IS THE ESTIMATED μ_t WITH $\rho_\mu = 0.50$. THE DASHED-DOTTED BLUE LINE IS THE ESTIMATED μ_t WITH $\rho_\mu = 0.00$.

We also check if our estimate of the persistence of the space sector share $\rho_s = 0.96$ is robust and fits our data well. Also, in this case, we have fixed the parameter to the following values

[2]The replication files of all the results in the SI appendix and the main text can be found on the authors' websites and at the following link: The-Macroeconomic-Spillovers-from-Space-Activity.

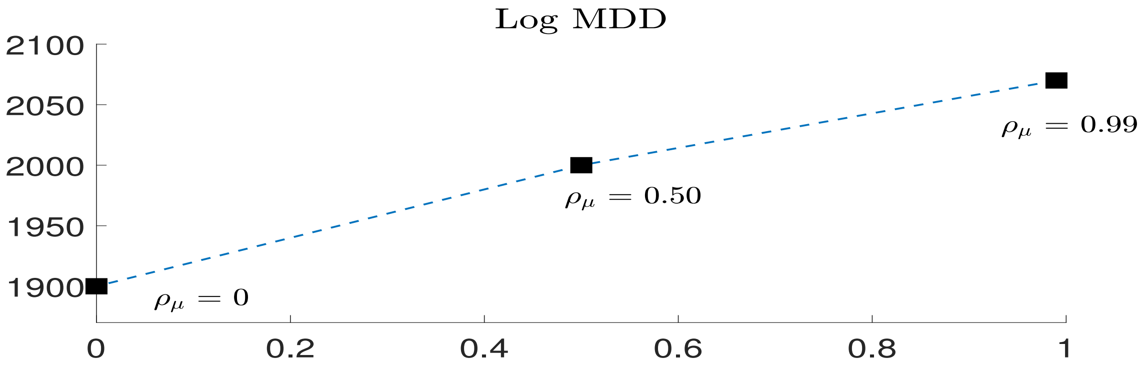

FIGURE S2. THE FIGURE REPORTS THE log MARGINAL DATA DENSITY (log MDD) FOR DIFFERENT VALUES OF ρ_μ.

$\rho_s = \{0.00, 0.50\}$ implying lower degrees of persistence. In Figure S3 we show the results for the log MDD associated with these different models. The figure shows that, also in this case, the data strictly prefer our benchmark estimate of ρ_s.

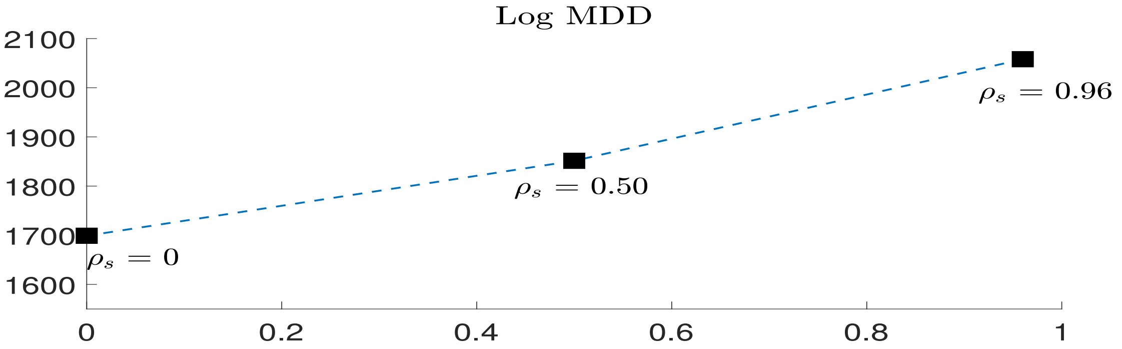

FIGURE S3. THE FIGURE REPORTS THE log MARGINAL DATA DENSITY (log MDD) FOR DIFFERENT VALUES OF ρ_s.

2) To further validate our results, we provide a robustness analysis for key calibrated parameters. Following standard practices in the literature on Dynamic Stochastic General Equilibrium (DSGE) models (see, among others, Smets and Wouters, 2007, Ireland, 2004, Christiano et al., 2005, and Herbst and Schorfheide, 2015), we have fixed these parameters to well-established values based on economic theory, as they are barely identifiable from the few series used in the estimation (see the Solution and Estimation Section of the main text). Taking all this into account, we show that changing the values of these parameters does not change the economic results of our paper. As we consider a space sector which has, to date, never been introduced into macroeconomic models, we specifically provide robustness with respect to the calibrated parameters of the space sector (α_s, ν_s, δ_{k_s}). In Figure S4, we show how the spillover of the space sector, μ_t, changes when we perturb these parameters from their benchmark values as reported in Table 1 of the main text. As shown in Figure S4 the movement in the spillover is robust to different values, including a lower or higher capital share parameter ($\alpha_s = \{0.25, 0.45\}$), a more elastic labor supply to the space sector ($\nu_s = \{0.50, 1\}$), and a higher depreciation rate of space sector capital ($\delta_{k_s} = 0.03$). Additional checks are reported

in the repository containing the replication codes[3] and show that the dynamics of the space sector spillover are robust even if the values of calibrated parameters in the core sector are changed. Of course, if we consider unrealistic values for calibrated parameters outside the range recommended by the literature, we get unreasonable results.

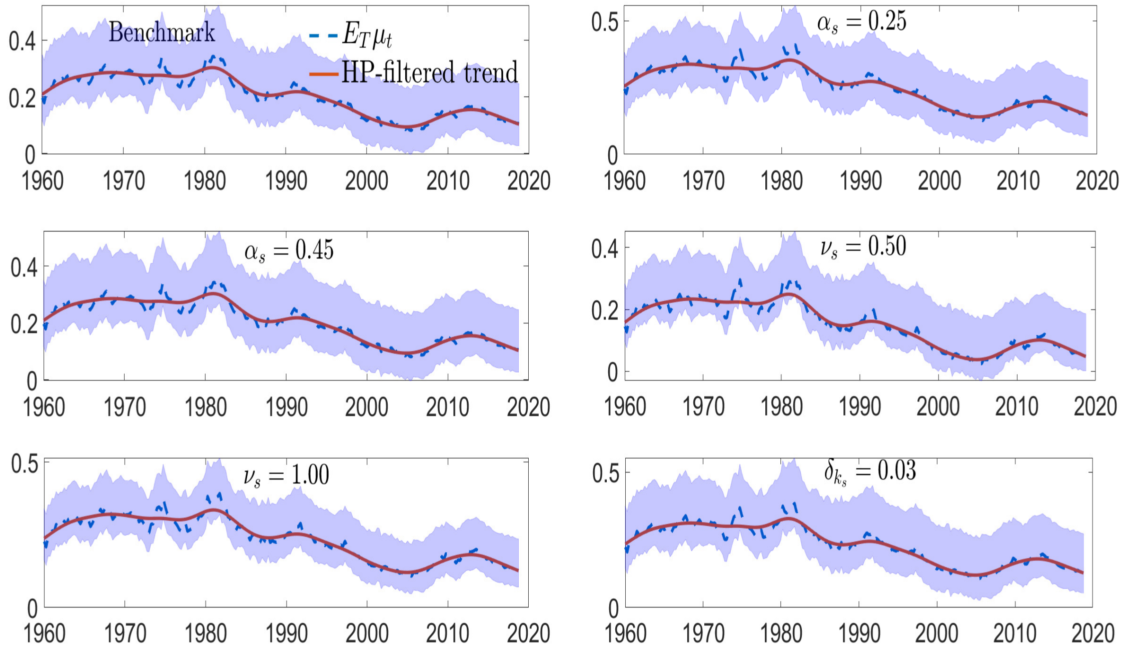

FIGURE S4. THE FIGURE REPORTS DIFFERENT ESTIMATES OF THE SPACE SECTOR SPILLOVER, μ_t, WITH DIFFERENT VALUES OF THE CALIBRATED PARAMETERS. THE PLOT IN THE UPPER LEFT REPORTS THE BENCHMARK MODEL. THE OTHER PLOTS REPORT RESULTS FOR $\alpha_s = \{0.25, 0.45\}$, $\nu_s = \{0.50, 1\}$ AND $\delta_{k_s} = 0.03$.

B. *Information Technology Sector*

To check whether the model tells us something about space in particular, we re-estimate the model by replacing the space industrial production data with the Information Technology (IT) production data. The series we consider is *Industrial Production: Equipment: Information Processing and Related Equipment* (code IPB52120S), freely available at `https://fred.stlouisfed.org/series/IPB52120SQ`. The series represents the level of activity in the industry that provides hardware, software, communication equipment and information equipment and it is available at monthly frequencies. To harmonize it with our quarterly data, we aggregate it at the quarterly frequency.

The model estimated with the IT sector produces different results than the model with the space sector. Figure S5 plots the spillover for the IT sector and shows that it has an upward trend from the beginning of the sample and does not decrease at the end, in accordance with the evidence of the high importance of IT in the last few decades.

[3]The results and the replication codes are available here: The-Macroeconomic-Spillovers-from-Space-Activity.

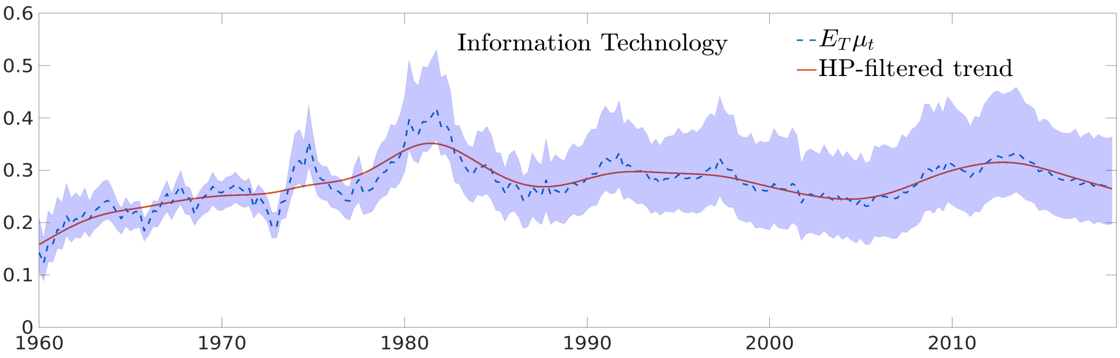

FIGURE S5. THE SPILLOVER FROM THE IT SECTOR ($\mathbb{E}_T\mu_t$, BLUE DASHED LINE) WITH THE HODRICK-PRESCOTT FILTERED TREND USING $\lambda^{HP} = 16000$ (HP-FILTERED, RED SOLID LINE). THE SHADED AREA REPRESENTS THE 68% CREDIBLE BANDS RELATED TO PARAMETER UNCERTAINTY.

C. *NASA plus Department of Defense*

Here we re-estimate the model by substituting the space industrial production data with the data of NASA plus the Department of Defense (DoD) space budgets. Unfortunately, the data has annual frequencies while our dataset is at quarterly frequencies. We transform the time series from annual to quarterly (temporal disaggregation) using the Chow-Lin method (Chow and Lin, 1971). In Figure S6 we report the results using NASA plus DoD data for the period 1960 to 2020. As the

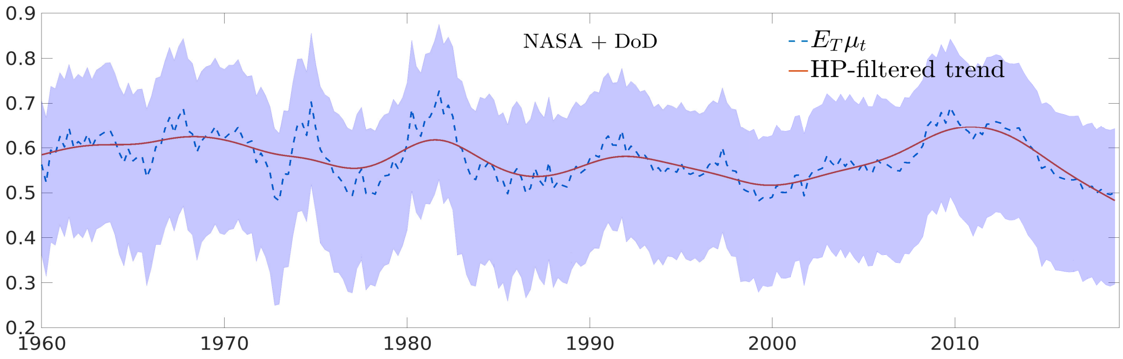

FIGURE S6. THE SPILLOVER FROM THE NASA+DOD SECTOR ($\mathbb{E}_T\mu_t$, BLUE DASHED LINE) WITH THE HODRICK-PRESCOTT FILTERED TREND USING $\lambda^{HP} = 16000$ (HP-FILTERED, RED SOLID LINE). THE SHADED AREA REPRESENTS THE 68% CREDIBLE BANDS RELATED TO PARAMETER UNCERTAINTY.

figure shows, the movement is qualitatively different from the model with the space sector industrial production. There is, moreover, high uncertainty in the estimation as the credible intervals (purple area) are larger in this case. This result is expected, as the temporal disaggregation cannot solve the problem of missing information at higher frequency (e.g., quarterly vs annual data).

D. Structural Vector Autoregression

The model

To investigate if we get qualitatively similar results using a less restrictive model, we now estimate a Structural Vector Autoregressive (SVAR) model. The SVAR by construction imposes fewer theoretical relationships among the economic variables. The starting point is to define a Vector Autoregression (VAR) given by:

$$y_t = \sum_{k=1}^{p} \mathrm{A}_k y_{t-k} + u_t, \qquad u_t \sim \mathcal{N}(0, \Sigma_u),$$

where p is the lag length, y_t stands for the vector of endogenous economic variables, A_k is a $k \times k$ coefficients matrix, and u_t is a Gaussian white noise with covariance matrix Σ_u. We consider five time-series over the period 1960:Q1 through 2018:Q4: real GDP, aggregate consumption, space production, the price index for private space fixed investment, and GDP deflator (see Table S1). All variables are introduced in log-levels and the lag length is set to four as we have quarterly series. We employ Bayesian techniques to estimate the reduced-form VAR model, for a detailed description see Antolín-Díaz and Rubio-Ramírez (2018).

Full name	Abbreviation	Frequency
Real Gross Domestic Product	GDP	Quarterly
Real Personal Consumption Expenditures	Consumption	Quarterly
Industrial Production: Aerospace Product and Parts	Space IP	Quarterly
Price Index for Private Space Fixed Investment	Space Price	Annual
GDP Implicit Price Deflator	Price Level	Quarterly

Table S1—Data Overview. Real GDP from BEA (retrieved from FRED): GDPC1. Consumption from BEA (retrieved from FRED): PCECC96. Industrial production is obtained from the Board of Governors of the Federal Reserve System (retrieved from FRED): IPG3364S. Price Index for Private Space Fixed Investment from BEA (retrieved from FRED): Y015RG3A086NBEA. For this series, we apply cubic spline interpolation to get the quarterly series. GDP deflator from OECD (retrieved from FRED): USAGDPDEFQISMEI.

The reduced-form innovations (u_t) in the previous model are cross-correlated, since the matrix Σ_u is not diagonal, implying that each innovation has contemporaneous effects on all macro variables. Consequently, this does not allow us to disentangle the effect of a specific innovation on the system. To solve this issue and give the model an economic interpretation, we need to identify the shocks that drive the economic variables (y_t) in a unique way, imposing restrictions on the driving errors (u_t). This is usually done by employing an identification matrix A^*, which brings to the SVAR:

$$\mathrm{A}^* y_t = \sum_{k=1}^{p} \tilde{\mathrm{A}}_k y_{t-k} + e_t, \qquad e_t = \mathrm{A}^* u_t \sim \mathcal{N}(0, \mathrm{A}^* \Sigma_u \mathrm{A}^{*'}),$$

where $\tilde{\mathrm{A}}_k = \mathrm{A}^* \mathrm{A}_k$, and e_t are the identified structural shocks that have an economic interpretation. To transform the errors of the reduced form, u_t, into structural shocks, $e_t = \mathrm{A}^* u_t$, it is necessary to place restrictions on the matrix A^*. Following Rubio-Ramírez et al. (2010), we start to impose traditional sign restrictions based on economic theory. In addition, we impose the narrative sign restrictions introduced in Antolín-Díaz and Rubio-Ramírez (2018), see Zeev (2018) for a related

approach. The main idea behind narrative sign restrictions is to boost identification, exploiting information from key historical events. To choose these historical events, we select the first mission for each major space program and constrain the historical decomposition of the space sector shock around these episodes, see Figure 2 in the main text. Specifically, our narrative is divided into two categories: early missions (Events 1-4) and latest missions (Events 5-6), to consider the potentially diverse effect of the space sector shock across the decades.[4]

We start imposing the sign restrictions required to identify our shocks, see Table S2. To purge the effects of the space sector shock from the confounding effects of other shocks, we also identify an aggregate demand shock and an aggregate supply shock in addition to the space sector shock. We assume that a space sector shock raises space production on impact. However, we are agnostic

	Space Shock	Demand Shock	Supply Shock
GDP	?	+	+
Consumption	?	?	?
Space IP	+	?	?
Space Price	?	?	?
Price Level	?	+	−

Table S2—Sign restrictions are imposed on impact. Symbols + and − refer to the direction of the response for the considered period of time. When being agnostic about the sign, the symbol ? is employed.

about the variable of interest: real GDP. One can also notice in Table S2 that we are silent about the response of prices in the space sector to a space sector shock, as we do not know whether this shock is demand-driven or supply-driven in its own industry.

We then add the narrative sign restrictions, where we assume that a space sector shock is the most important contributor to the unexpected changes observed in space industrial production. The narrative restriction is first imposed on the set of the early large public missions (Events 1-4) and then imposed on the set of the last two private initiatives (Events 5-6). In this way, we can study the different natures of space sector shocks before and after private companies entered the space race.

Results

Figure S7 plots the IRFs to a space sector shock. The blue solid line is the IRF that uses the narrative sign restriction applied to the early events (*Events 1-4* described in Figure 2 in the main text). When we use the narrative restriction around these events, we find that real GDP persistently and significantly increases upon impact by around 0.5% and remains elevated for many quarters at values larger than 1% (similar to the response of consumption). The red dashed line in Figure S7 shows, instead, the response including the narrative sign restriction applied only to the two most recent initiatives of Figure 2: *Event 5* (2008:Q3) – SpaceX Falcon 1's first successful orbital launch of any developed rocket under a partnership between NASA and a private company, and *Event 6* (2016:Q2) – the successful sub-orbital flight and landing of a reused booster (New Shepard 2) by Blue Origin. These events represent major contributions to the space industry. However, they have not yet achieved new major exploration milestones, as was the case for the early public space programs. Similarly to the results from the model in the main text, the SVAR evidence suggests that while the effects of space sector shocks on the economy were positive and significant in the

[4]We contemplate that the exogeneity of space sector shocks is guaranteed, given that the space missions were carried out independently of the economic conditions at each considered period. For example, in 2008:Q3 SpaceX achieved its first orbital launch (Falcon 1) and, at the same time, the US economy was in a profound downturn.

early stages of space activity, they appear to be less economically relevant in recent years. As the SVAR model imposes fewer theoretical relationships among the economic variables (see Antolín-Díaz and Rubio-Ramírez, 2018), the GDP response to a space sector shock is qualitatively similar to the model in the main text, yet with larger uncertainty.

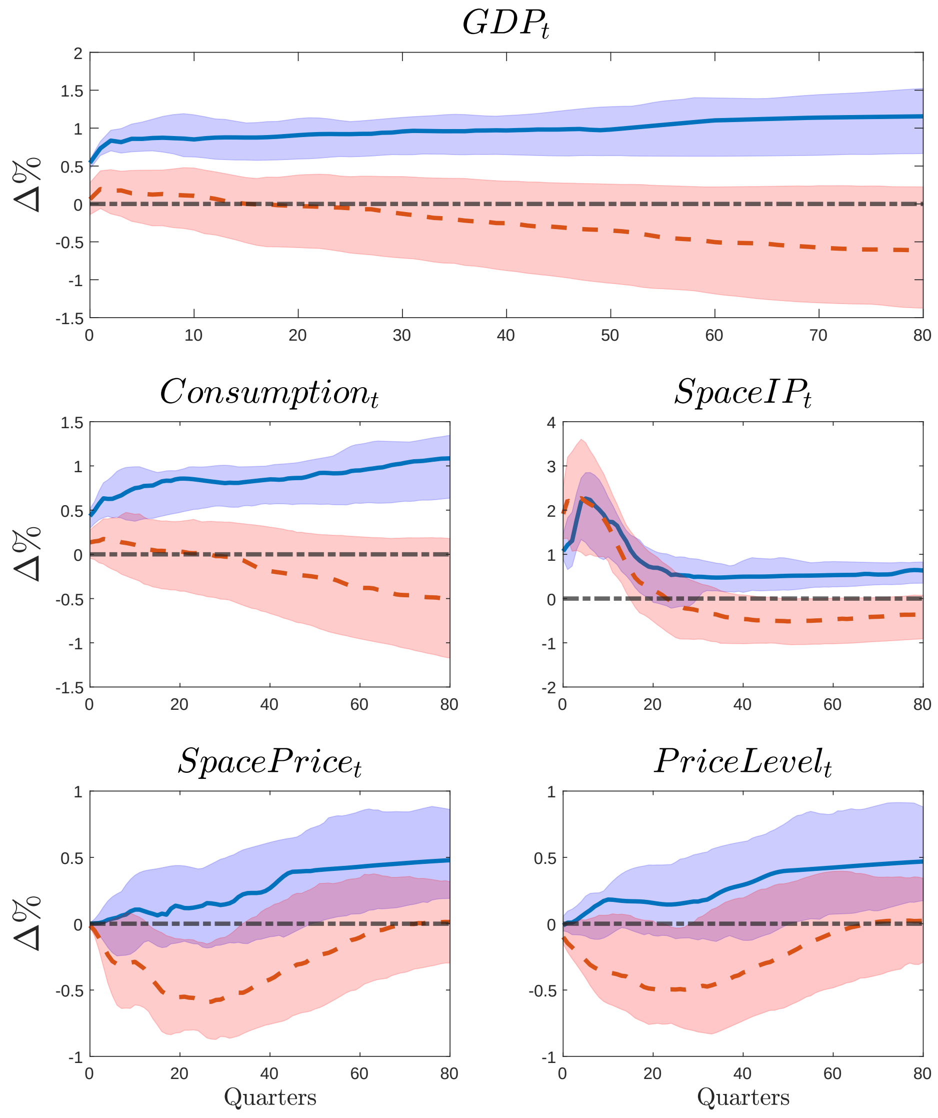

Figure S7. IRFs to one standard deviation space sector shock: early vs. recent initiatives. The blue solid lines represent the IRFs computed with the narrative sign restrictions applied to the early four events. The red dashed lines show the IRFs related to the two more recent events. The IRFs are expressed as percentage deviations with respect to the case where no shock realizes. Each entry shows the median and the 68% confidence bands.

REFERENCES

Adjemian, S., Bastani, H., Juillard, M., Karamé, F., Mihoubi, F., Mutschler, W., Pfeifer, J., Ratto, M., Rion, N., and Villemot, S. (2022). Dynare: Reference Manual Version 5. Dynare Working Papers 72, CEPREMAP.

Antolín-Díaz, J. and Rubio-Ramírez, J. F. (2018). Narrative Sign Restrictions for SVARs. *American Economic Review*, 108:2802–2829.

Chow, G. C. and Lin, A. L. (1971). Best Linear Unbiased Interpolation, Distribution, and Extrapolation of Time Series by Related Series. *The Review of Economics and Statistics*, 50:372–375.

Christiano, L. J., Eichenbaum, M., and Evans, C. L. (2005). Nominal Rigidities and the Dynamic Effects of a Shock to Monetary Policy. *Journal of Political Economy*, 113:1–45.

Geweke, J. (2005). *Contemporary Bayesian Econometrics and Statistics*. Wiley, New York, USA.

Herbst, E. P. and Schorfheide, F. (2015). *Bayesian Estimation of DSGE Models*. Princeton University Press.

Ireland, P. N. (2004). A Method for Taking Models to the Data. *Journal of Economic Dynamics and Control*, 28(6):1205–1226.

Koop, G. (2003). *Bayesian Econometrics*. John Wiley and Sons Ltd, England.

Rubio-Ramírez, J. F., Waggoner, D., and Zha, T. (2010). Structural Vector Autoregressions: Theory of Identification and Algorithms for Inference. *Review of Economic Studies*, 77:665–696.

Smets, F. and Wouters, R. (2007). Shocks and Frictions in US Business Cycles: A Bayesian DSGE Approach. *American Economic Review*, 97(3):586–606.

Zeev, N. B. (2018). What Can We Learn about News Shocks from the Late 1990s and Early 2000s Boom-Bust Period? *Journal of Economic Dynamics and Control*, 87:94–105.
